# Quantifying and Mapping the Supply of and Demand for Carbon Storage and Sequestration Service from Urban Trees

**DOI:** 10.1371/journal.pone.0136392

**Published:** 2015-08-28

**Authors:** Chang Zhao, Heather A. Sander

**Affiliations:** Department of Geographical and Sustainability Sciences, University of Iowa, Iowa City, Iowa, United States of America; DOE Pacific Northwest National Laboratory, UNITED STATES

## Abstract

Studies that assess the distribution of benefits provided by ecosystem services across urban areas are increasingly common. Nevertheless, current knowledge of both the supply and demand sides of ecosystem services remains limited, leaving a gap in our understanding of balance between ecosystem service supply and demand that restricts our ability to assess and manage these services. The present study seeks to fill this gap by developing and applying an integrated approach to quantifying the supply and demand of a key ecosystem service, carbon storage and sequestration, at the local level. This approach follows three basic steps: (1) quantifying and mapping service supply based upon Light Detection and Ranging (LiDAR) processing and allometric models, (2) quantifying and mapping demand for carbon sequestration using an indicator based on local anthropogenic CO_2_ emissions, and (3) mapping a supply-to-demand ratio. We illustrate this approach using a portion of the Twin Cities Metropolitan Area of Minnesota, USA. Our results indicate that 1735.69 million kg carbon are stored by urban trees in our study area. Annually, 33.43 million kg carbon are sequestered by trees, whereas 3087.60 million kg carbon are emitted by human sources. Thus, carbon sequestration service provided by urban trees in the study location play a minor role in combating climate change, offsetting approximately 1% of local anthropogenic carbon emissions per year, although avoided emissions via storage in trees are substantial. Our supply-to-demand ratio map provides insight into the balance between carbon sequestration supply in urban trees and demand for such sequestration at the local level, pinpointing critical locations where higher levels of supply and demand exist. Such a ratio map could help planners and policy makers to assess and manage the supply of and demand for carbon sequestration.

## Introduction

Ecosystem services are the benefits people obtain from ecosystems upon which human well-being largely depends [1]. A wide variety of services exist, falling generally into four categories: regulating services (e.g., pollination, climate regulation, air quality regulation, water purification), provisioning services (e.g., food production, biomass, freshwater, mineral resources), supporting services (e.g., nutrient cycling, primary production, soil formation) and cultural services (e.g., recreation, aesthetic views, cultural heritage) [1]. Despite their wide recognition in biological conservation and natural resource management, ecosystem services are not well-integrated in land-use planning and management [2]. One of the key hurdles to considering ecosystem services in decision making is the present incomplete understanding of how services are delivered from ecosystems to social systems (i.e., the supply side of ecosystems services), and the extent to which human well-being relies on ecosystem services (i.e., the demand for ecosystem services), as well as the relationship between these two sides.

Improving this understanding requires us to distinguish among the aspects of ecosystems that influence service delivery (e.g., ecosystem structures, ecosystem functions) and ecosystem service potential, flow, and demand [3]. Ecosystem structures are the set of components that make up an ecosystem, while ecosystem functions are the processes and cycles that occur in an ecosystem [4]. For example, with respect to carbon storage and sequestration, an urban forest ecosystem may be characterized in terms of its autotrophs, heterotrophs, water and soils through which ecological functions such as photosynthesis and carbon cycling and nutrient cycling occur. While ecosystem structures and functions are essential to support overall ecosystem integrity and resilience, they may not directly contribute to human well-being. For example, water uptake by plants, infiltration of soil, soil stabilization and nutrient cycling maintain the integrity and resilience of riparian systems, but, depending on the system considered, may not supply services (e.g., flood mitigation, provision of clean water for drinking) unless these products are actually used by humans. Ecosystems services thus are the products of ecosystem structures and functions that actually do contribute to human well-being [5,6].

The notion of human dependence on nature implies that ecosystem services do not exist without beneficiaries, as goods and benefits must be either directly or indirectly consumed, used, or desired by humans [7]. Some researchers have distinguished between ecosystem service potential and flow. While ecosystem service potential is commonly referred to as the total capacity of ecosystems to generate services [3,8], the term ecosystem service flow is much more ambiguous, referring either to the spatial connections between service provisioning areas (SPA) and service benefiting areas (SBA) [9–11], or the actual production or use of ecosystem services at a given location [3,12,13]. Note that potential and flow as defined above may also be referred to as supply and demand, although with slightly different meanings. While supply refers to the type and quantity of ecosystem services associated with the geophysical and ecological characteristics of ecosystems [14], demand for ecosystem services reflects those services actually consumed or desired by beneficiaries [11]. The distinction between these terms is crucial for selecting appropriate methods and indicators for quantifying and mapping the different dimensions of ecosystem services and provides clarity in the use of associated products (e.g., statistics, maps) that support decision making. Therefore, to improve our understanding of ecosystem services and to better incorporate this concept in policy making, it is necessary to capture the full ecosystem service dynamic moving from service supply to demand by assessing various aspects of ecosystem service delivery.

Following the completion of the MEA in 2005, several international initiatives called for attention to the economic benefits of natural capital and biodiversity (e.g. The Economics of Ecosystems and Biodiversity, Intergovernmental Platform on Biodiversity and Ecosystem Services) [1]. In response the number of studies measuring the supply of ecosystem services grew exponentially. These studies use various methods to assess ecosystem service supply, including empirical models, process-based models (e.g. hydrological models, climate models), GIS-based approaches, expert knowledge, and decision-support tools (e.g. Integrated Valuation of Ecosystem Services and Tradeoffs, InVEST; Artificial Intelligence for Ecosystem Services, ARIES; Global Unified Model of the BiOsphere, GUMBO) [15–20]. While some of these tools implicitly consider demand via their economic valuation components (e.g., InVEST) or explicitly account for ecosystem service flows from SPA to SBA via simulations (e.g. ARIES), modeling approaches that assess the demand side of ecosystem services remain rare [5]. The majority of ecosystem service studies focus solely on service supply and lack a systematic, integrated consideration of demand, often as a result of data limitations with respect to human needs and well-being. For example, potential recreational services may be estimated for a forest based on landscape characteristics, but the actual ability of populations to access and enjoy these services may not be considered. Such a lack of consideration for ecosystem service demand creates a knowledge gap that impacts the quality of management and policy decisions made, in this example, regarding forest management.

Given the importance of considering both the supply and demand sides of ecosystem services in decision making, several conceptual frameworks for analyzing ecosystem services in an integrated manner have been produced in recent years [12–14]. Although the literature that examines both the supply and demand of ecosystem services has recently grown [9,21–25], studies which specifically distinguish among ecosystem service supply and demand remain rare. Demand in existing studies is commonly conceptualized from three perspectives. Firstly, demand for provisioning services may be quantified as the use or consumption of those services. In such cases, population size and average consumption rates are often combined to produce indicators of demand. This, for example, was the case in a recent study that calculated demand for the energy provisioning service based upon statistical data on the energy consumption per unit area associated with different land cover types [26]. Secondly, for most cultural services, demand has been assessed based largely on social preference or on ecosystem service valuation [27–29]. For example, participatory mapping of ecosystem service demand via interviews has been used to assess preferences for and perceptions of various cultural services at the community level, recognizing that ecosystem service demand is driven by lifestyle choices, demographic characteristics and cultural beliefs [28]. Lastly, demand for supporting and regulating services is most often expressed in terms of the social desire or need to reduce risks or increase service benefits [22,24,25,30]. For example, demand for flood regulating services may be quantified using the potential vulnerability of assets and monetary risk associated with loss as indicators [22]. However, estimating demand for maintaining desirable environmental conditions is often challenging because doing so requires information about the need for risk reduction and protection. In most cases this entails quantitative modeling of many factors, including potential risks, levels of exposure and degrees of social vulnerability [11].

Quantification of the demand side of regulating services is particularly challenging because these services are neither directly used nor consumed. In addition, demand for regulating services is often underestimated by society due to the complexity of the natural and social processes associated with their delivery. Process-based models of vulnerability to risk and accessibility to service supply are often used to determine the degree of demand for regulating services from a risk-reduction perspective. For example, previous studies have quantified flood regulation service demand based on the presence of a flood hazard, possibility of exposure and vulnerability of assets [22,25]. Similarly, exposure to natural hazards, people and assets has been estimated to assess demand for coastal protection services [31]. This risk reduction perspective is less desirable for quantifying demand for carbon storage and sequestration. This is because climate change, the major risk that can be reduced or avoided through carbon storage and sequestration, is difficult to explicitly model and may further be perceived as a multiplier of other risks (e.g., reduced water availability, intensified urban heat islands). Given that this risk is entangled with negative consequences that are linked to other ecosystem services (e.g., water production, microclimate regulation), directly modeling demand for carbon storage and sequestration based on risk could lead to double-counting issues.

Proxy-based methods offer an alternative to the use of process-based methods to model desire for reducing the risk of climate change. These methods use a proxy, or indicator, to identify ecosystem service delivery or demand levels. Proxies are useful when the state and trend of targeted ecosystem services are not directly monitored or measurable. Previous studies have used proxies for mapping the distribution of ecosystem service supply (e.g., land use types, net primary productivity, ecosystem components and functions) [32], and they may also be used in estimating demand in a way that avoids many of the issues related to double-counting discussed above.

We present an approach to assessing ecosystem service supply and demand in a spatially-explicit manner focusing on carbon storage and sequestration provided by urban trees. In so doing, we seek to improve our understanding of the balance between supply and demand for this service and our ability to consider this service in local land-use decision making. Urban carbon storage and sequestration provides a highly policy-relevant arena for exploring the balance between service supply and demand. Although climate change mitigation via carbon storage and sequestration is delivered globally, understanding the degree of spatial mismatch between supply and demand for this service at the local level remains critical to its management because current global policies fail to effectively regulate local carbon emissions. Thus, setting policies which seek to mitigate climate change, for example those aimed at producing carbon-neutral cities, requires us to move to regional and local spatial scales. These scales are more relevant to local policy makers as they provide spatially-explicit estimates of the supply of and demand for carbon storage and sequestration within their jurisdictions. Such estimates could thus facilitate carbon balance assessments for cities and improved policy making.

For the purposes of this study, we define an ecosystem service flow as the actual services delivered from SPA to SBA. Given that carbon storage and sequestration is “used” to mitigate climate change, the supply of this service constitutes a flow from SPA to SBA. We thus refer to this flow as a “supply”. We also distinguish between ecosystem service supply and demand by defining demand for carbon sequestration as the benefits desired or expected by beneficiaries which, in return, is driven by factors including biophysical supply, population size, consumption patterns, cultural perceptions and valuation of ecosystem services [3,11].

Our approach begins with the assessment and mapping of the supply side of this service, conceptualizing supply of carbon storage as the total amount of carbon stored in urban trees and supply of carbon sequestration as the amount of carbon sequestered by trees annually. We then assess and map demand using a proxy for the social desire for this service, quantifying benefits expected by society as a function of local annual anthropogenic CO_2_ emissions. To facilitate comparison of supply and demand for carbon storage and sequestration and to identify critical locations where imbalances in supply and demand occur, we lastly conduct a spatially-explicit supply-to-demand ratio analysis. In this way, our approach facilitates not only identification of the general carbon storage and sequestration service supplied by urban trees and the demand for this service across the study area, but also the specific locations to which management and policy might be targeted to improve the provision of this service. As such, our approach could enable policy makers to clearly target land use planning and management to locations where it would most increase service supply or where critical actions to reduce demand are needed.

## Overview of Carbon Storage and Sequestration Modeling

Carbon storage and sequestration provided by trees in urban areas has been addressed in a number of studies. Field-collected data on urban forest structure (e.g., species; diameter at breast height, dbh; height; crown width) are most commonly used to estimate the supply of carbon storage and sequestration provided by urban trees [33–37]. The frequently-used i-Tree Eco model, which uses field-generated tree inventory data to estimate ecosystem services from urban trees, has been applied in numerous US cities to measure carbon storage and sequestration [38]. For instance, urban trees in Brooklyn, New York were estimated to store approximately 172,000 metric tons of carbon with a value of $3.5 million [39] while urban trees on public lands in Corvallis, Oregon were estimated to store about 71,000 metric tons valued at $1.45 million [40].

Remote sensing-based methods are also used to estimate carbon storage and sequestration by trees and can be helpful where forestry applications require quickly-updated data over larger extents. A number of remote-sensing technologies are now available to expedite the estimation of urban forest structure and corresponding carbon storage and sequestration [41]. Compared to passive sensors which excel at detecting the two-dimensions of ground features, terrestrial and airborne Light Detection and Ranging (LiDAR) are particularly promising, allowing for examination of the vertical structures of natural and built environments with high precision to facilitate assessments of aboveground forest biomass and bioenergy [42,43].

While studies that use these methods quantify the supply side of carbon storage and sequestration by urban trees, they do not compare this with demand for this service, making it difficult to understand ecosystem service dynamics and the relationship between service delivery and human well-being. Some studies, however, have compared estimated supply to demand [44,45]. For example, Baró et al. (2014) applied the i-Tree Eco model to quantify the biophysical supply of carbon storage and sequestration within the Barcelona metropolitan area in Spain and compared it to carbon emissions to assess the relative contribution of this service to climate change mitigation under targeted environmental policies [45]. These authors, however, did not examine the degree of balance in the supply and demand of carbon storage and sequestration in a spatially-explicit manner, but focused on the metropolitan area as a whole and did not identify key locations of provision and demand for this service.

As noted above, forest inventory data together with field sampling are generally used for biomass estimation. These data are often available at a coarse grain over large extents and very few fine-grained datasets exist for local and regional extents [43]. Urban tree inventory data are particularly lacking because urban trees are not systematically monitored by government agencies and field data collection is expensive, labor-intensive and time consuming [46]. Where these data exist, they are often limited to selected sites and may not be representative of urban forest characteristics across the larger region. Interpolation techniques can be used to overcome this problem, but, because of the heterogeneous nature of urban forests, are likely to introduce error to the modeling processes and increase uncertainty in carbon storage and sequestration estimation. As described above, the i-Tree Eco model is widely used for quantifying ecosystem services associated in urban trees. Since detailed urban forest data, such as species, dbh, total height, crown width and tree health condition are needed to implement carbon calculations using this model [45], it suffers from the same disadvantages as general field-data based methods discussed above.

Remotely-sensed LiDAR data may be analyzed to provide a faster, efficient and reliable way to identify urban forest characteristics for the assessment carbon storage and sequestration by urban trees. Airborne laser scanning technology enables LiDAR to detect numerous ground features over a large spatial extent. LiDAR emits an intense and focused laser pulse and measures the elevation of ground features by computing the time it takes for the sensor to detect laser returns, the angle at which the laser pulse is emitted, and the sensor location. Multiple laser returns are typically used to characterize both the horizontal and vertical structure of trees [43]. A first return is typically generated from the uppermost portion of the tree canopy, followed by multiple less-intense returns down through the canopy and a last return of the underlying terrain. Since LiDAR data sources are publicly available in many municipalities, using LiDAR datasets to assess urban forest structure can facilitate analyses of the supply of carbon storage and sequestration service provided by urban trees. This is particularly useful in dense urban environments because it facilitates efficient biomass and carbon calculations without the time, economic, and labor expenditures associated with intensive field sampling. LiDAR technology also has great potential to be used to generate reliable estimates of the supply of carbon storage and sequestration by trees because of its high accuracy in extracting three dimensional attributes of trees which might be obscured by cloud or shadows of other structures in aerial photography (e.g., height, crown width, dbh).

## Methods

**Fig 1:**
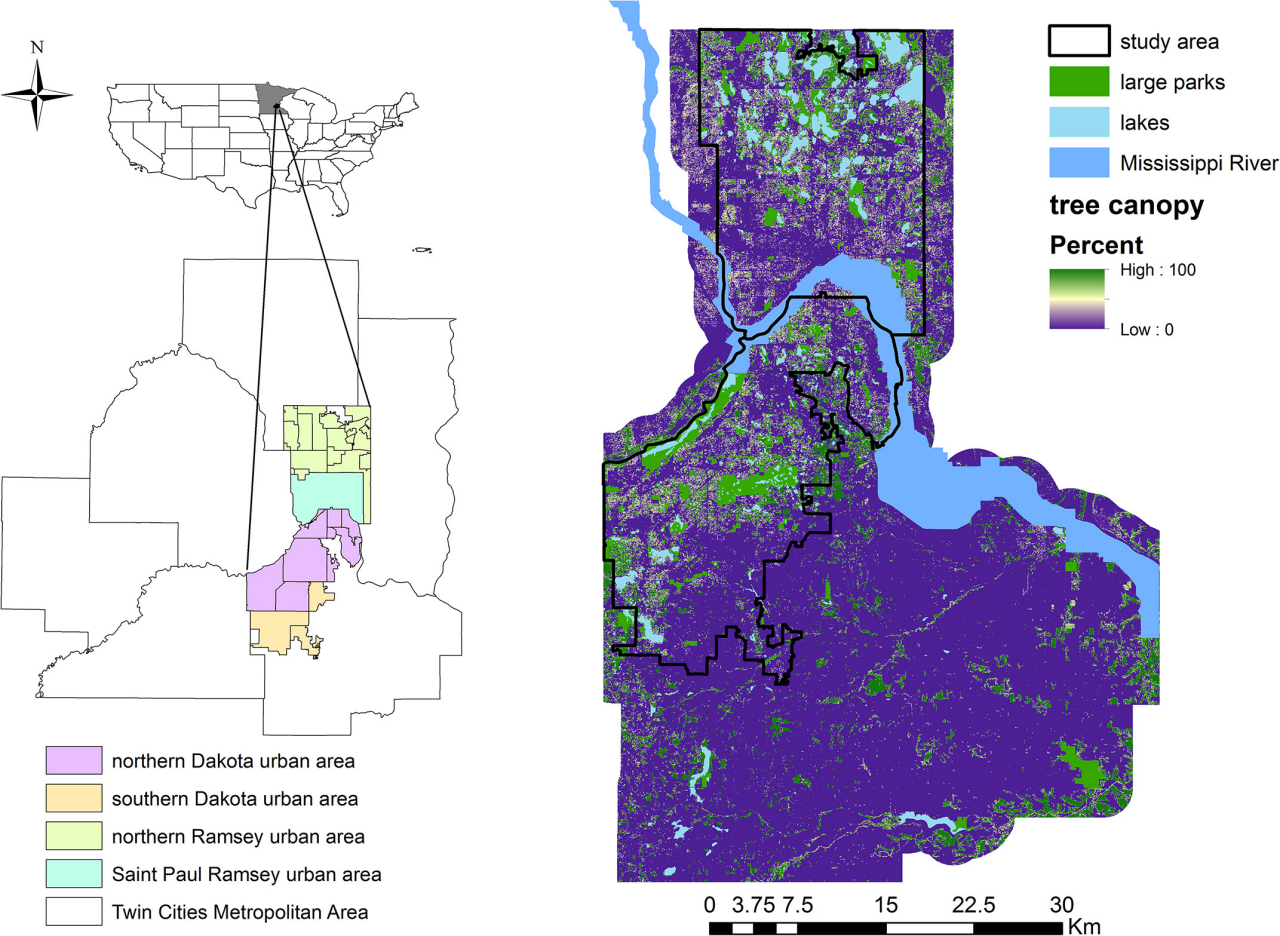
Location of the study area, urbanized areas of Dakota and Ramsey County, MN, including tree canopy coverage, parks and water body locations. This study area is divided into four regions (i.e., northern Dakota, southern Dakota, northern Ramsey, St. Paul Ramsey) to coincide with the regions for which tree abundance data used in the study were collected [47].

**Fig 2:**
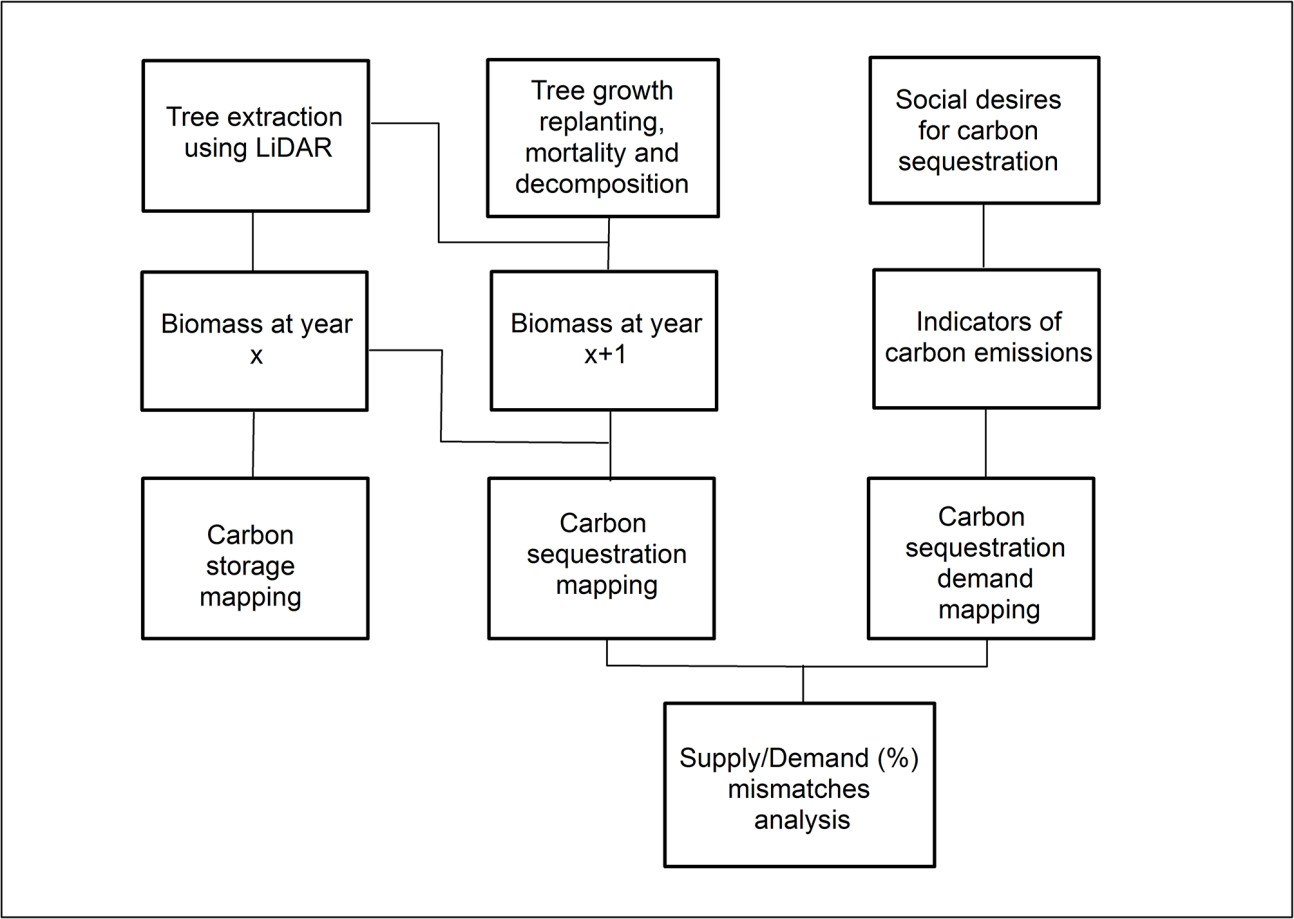
Methodological approach to the assessment and mapping of the supply and demand for carbon storage and sequestration.

We develop a method for quantifying, mapping and comparing the supply of and demand for carbon storage and sequestration by urban trees. We implement this method in our study area, a portion of the Twin Cities Metropolitan Area (TCMA) of Minnesota, USA (Fig 1). Our basic approach involves four steps: (1) estimating and mapping the supply of carbon storage using LiDAR technology and allometric models, (2) estimating and mapping the annual supply of carbon sequestration based on carbon gains from biomass growth and carbon loss from tree mortality and decay, (3) estimating and mapping demand for carbon sequestration based on the intensity of CO_2_ emissions by a set of human activities under the assumption that demand for carbon sequestration service increases with anthropogenic carbon emissions, and (4) analyzing the relationship between the supply of this service (carbon sequestration) and the demand for it (Fig 2).

### A. Study area

We illustrate this integrated approach using the urbanized areas of Dakota and Ramsey County in the TCMA (Fig 1). This study area includes an urban land area of 856 km^2^ with a population of 359,365 and is comprised of 35 cities and towns, among which the state capital, St. Paul, is the second-most populous city in Minnesota. This urban land area was defined using the Urbanized Extents defined by the US Census and includes areas that are predominately, but not entirely, urban in their character. For the 2010 Census, an urban area comprises densely settled census tracts that have at least 2,500 people, along with adjacent land containing non-residential urban land uses as well as other land use types with low population density (e.g. forests, agriculture, non-developed land) [48]. In this study area, Dakota County is characterized by a mixture of urbanized land and forest reserves with dense tree coverage in the north, while southern portions of the county are dominated by agriculture with sparse trees. Similarly, northern Ramsey is mainly characterized by natural landscapes with dense tree coverage, while southern Ramsey is dominated by dense developed areas with sparser, street trees.

Because urbanization patterns in this study area are typical of those in most of the US, this location provides a representative location for quantifying and mapping supply and demand for urban carbon storage and sequestration from urban trees, given both the high number of urban trees and beneficiaries who receive their services in this location.

### B. Tree extraction with LiDAR data

We retrieved 1175 tiles containing LiDAR point-cloud data collected in November, 2011, with a vertical accuracy of 5 cm for Ramsey County and 10.8 cm for Dakota County from the Minnesota Geospatial Information Office (MnGeo) [49]. This high-density dataset contains 8 points/m^2^ for Ramsey and northern Dakota County and 2 points/m^2^ for southern Dakota County. These data contain information on return number and number of returns, intensity of return signal, coordinates, scan direction and point cloud classifications, which are useful for tree feature extraction. This dataset uses oversampling techniques and has a vertical accuracy of up to 50 cm and a horizontal accuracy of up to 30 cm.

We used the LiDAR Analyst 5.0 extension [50] of ArcGIS 10.1 [51] to identify biophysical attributes of trees in the study area that are relevant to the estimation of biomass. This proprietary automated feature extraction-based tool (AFE) uses polygon decimation techniques, including shape-matching and machine-learning approaches, for LiDAR analysis [52]. LiDAR processing can provide two distinct surfaces: a Digital Elevation Model (DEM) which contains bare earth elevations, and a Digital Surface Model (DSM) which contains elevation information for tree canopy and buildings as well as other features (e.g., shrubs, bridges, elevated roadways). Extraction processes for trees require both an accurate DEM and DSM. Typically, a DSM is modelled by removing any point clouds belonging to the ground. Buildings also need to be correctly identified because they are critical for effectively modeling and displaying trees on an accurate DSM. The tree-extraction process thus begins with the extraction of bare earth and buildings from a sample tile, followed by adjustment of the extraction parameters controlling the detection process to ensure accurate feature extraction. In our analysis, default parameter settings were sufficient for extracting bare earth elevations as no significant distortion or raised features were observed. We classified LiDAR point clouds to ground and non-ground through bare earth extraction. A point-cloud-based method was selected for building extraction. We isolated and filtered trees based upon the bare earth DEM and corrected building features using a fixed-window search often applied to the analysis of dense urban forests. We set a minimum tree height of 3 m and typical tree height of 20 m, since this identified most tree and forest features. This presents a limitation as a tree under 3 m tall can contain a large amount of biomass. Nevertheless, considering the tendency for LiDAR to omit understory vegetation, particularly in dense urban areas, our 3 m threshold for tree extraction ensures high parameter prediction accuracy. This process produced a point shapefile, representing individual trees with their attributes, including tree height, crown width and dbh. Once we set workflow and extraction parameters with the sample tile in this way, we used batch processing to extract tree features from other tiles in the dataset based on the AFE algorithm.

Since tree extraction is based on a shape-matching approach, ground features similar to trees may be misclassified as trees. Infrared beams emitted by LiDAR sensors also tend to be absorbed by water and return very weak to no signals, resulting in poorer extraction performance. To validate LiDAR results, we visually compared the extracted results with a 2012 orthophoto of the TCMA seven-county metropolitan area from the Minnesota Department of Natural Resources (MNDNR). We deleted misclassified trees on water and roof tops, and trees that were actually water towers or other round features. Tree records from the MNDNR Minnesota Native Big Tree registry [53] were used to help eliminate trees of abnormal height (>40 m), dbh (>3.187 m) or crown width (> 42.672 m).

To assess tree-extraction accuracy, we collected tree attribute measurements from the orthophotograph mentioned above, then selected 50 trees distributed evenly across the study area, and measured their crown width using ArcGIS 10.1. These trees had clear crown shapes to minimize measurement error. We fitted a linear regression model using the resulting dataset and calculated root mean squared error (RMSE) using our measured values and the LiDAR Analyst results to determine the accuracy of the LiDAR metric in predicting tree attributes.

### C. Estimation of carbon storage

Estimation of tree dry-weight biomass is of great interest since such information indicates carbon storage and could enable policy makers to track carbon dynamics in biomass [54]. We used taxon-specific biomass equations to estimate above-ground biomass following the work of Jenkins et al. (2004) which used allometric equations and conversion factors to transfer above-ground biomass to dry-weight biomass based on dbh [37,54]. We based our calculations on existing field datasets that identified the relative abundances of common species by land use intensity and location in the study area (Table 1) [47,54]. Based upon these sources, we classified common tree species in the study area into seven classes: soft maple (*Acer negundo*, *Acer saccharinum*, *Acer pensylvanicum* and *Acer rubrum*) /birch (*Betula spp*.), aspen/alder/cottonwood/willow (*Populus*, *Alnus* and *Salix spp*.), hard maple (*Acer saccharum*, *Acer plantanoides* and *Acer nigrum*)/oak (*Quercus spp*.)/hickory (*Carya spp*.)/beech (*Fagus spp*.), other hardwood, cedar (*Chamaecyparis spp*.)/larch (*Larix spp*.), spruce (*Picea spp*.) and pine (*Pinus spp*.). The tree species groupings for this analysis present a possible limitation as differences in biomass carbon storage may exist among species in these groups and as tree composition in urban areas is normally diverse and may contain species that are not well-represented by this classification system. Nevertheless, this is the most specific grouping system possible given data and model limitations and is sufficient to illustrate the application of our methodology. Additionally, although calibrating allometric models for specific species is of interest, our current understanding of the relationship between tree biophysical attributes and biomass at a single-species level is insufficient to support such calibrations [46]. Moreover, allometric models for different species have very similar shapes, thus causing minimal differences in biomass predictions among species.

**Table 1:** Relative abundance of tree species groups by region and land use and parameter values for biomass estimation in Dakota and Ramsey urban areas, MN.

Species	Relative abundance (%)^1^					*β*_*0*_	*β*_*1*_	Ave. dbh growth rate (cm/yr)
	Developed land				Undeveloped land			
	N. Dakota	S. Dakota	N. Ramsey	St. Paul Ramsey				
mb^2^	12.82	18.23	17.47	18.17	11	-1.9123	2.3651	0.152
aa^3^	6.52	6.99	3.72	0	13	-2.2094	2.3867	0.406
mo^4^	5.19	2.95	0	0	14	-2.0127	2.4342	0.328
oh^5^	63.09	54.91	68	76.64	64	-2.4800	2.4835	0.282
cl^6^	8.29	2.73	5.35	5.19	0	-2.0336	2.2592	0.185
sp^7^	4.09	14.19	5.46	0	0	-2.0773	2.3323	0.348
pi^8^	0	0	0	0	1	-2.5356	2.4349	0.345

^1^ percent of total trees represented by each species group

^2^ soft maple/birch

^3^ aspen/alder/cottonwood/willow

^4^ hard maple/oak/hickory/beech

^5^ other hardwood

^6^ cedar/larch

^7^ spruce

^8^ pine, *β*_*0*_ and *β*_*1*_ are parameters used in biomass equations for estimating total aboveground biomass for hardwood and softwood species in the United States.

Table was produced based upon previous studies [47,54,71].

Since LiDAR processing does not provide species information, we identified likely tree species for the trees characterized by LiDAR processing based upon the relative abundances of common species estimated for the study area in previous studies (Table 1) [47]. In so doing, we first classified trees as occurring on developed or undeveloped land and by location. Then, for each group and location, we randomly sampled a percentage of trees corresponding to the relative abundance estimated by the field studies for that group and location and assigned them to the specified species group. We repeated this for each species group and location until all trees were assigned a species group. Once tree species were assigned, we applied the following equation to calculate above-ground biomass for each tree:

$$B=Exp(\beta_{0}+\beta_{1}\;*\;\ln(dbh))$$ (1)

Where

*β*_*0*_, *β*_*1*_ is based on species type (Table 1)
*B* = total aboveground biomass (kg) for trees 2.5 cm and larger in dbh

Although the root-to-shoot ratio varies somewhat from species to species, we assumed a fixed root-to-shoot ratio of 0.26 for all species groups and converted above-ground biomass to whole-tree, dry-weight biomass following past studies [35,55]. Total tree dry-weight biomass was multiplied by 0.5 to calculate the total stored carbon in trees which approximates the proportional mass of carbon in trees [37,56–58].

### D. Estimation of carbon sequestration

Our estimates of carbon sequestration firstly consider the probability of tree mortality. To generate a subset of trees surviving to the next year, we used tree mortality rates by dbh from a study of Chicago's urban forest ecosystem [34]. Although this study dates from 1994 and occurred outside of our study area, it is appropriate for mortality estimation in this analysis since our study area shares the same ecoregion (Eastern broadleaf forest) and has similar land use and climate conditions and tree composition [59]. Additionally, no other recent study of which we are aware exists for this region. This study estimated annual tree mortality rates of 2.1% for trees between 16 and 46 cm dbh, 2.9% for trees between 47 to 61 cm dbh, 3.0% for trees between 62 and 76 cm dbh, and 5.4% for trees greater than 77 dbh. Based upon this information, we randomly sampled trees in each dbh class to identify an appropriate percentage of trees surviving to the next year for use in estimating sequestration rates.

Our consideration of decomposition is based on best evidence and is conservative. Tree decomposition rates vary with their physical characteristics, location and environment. Within the city of St. Paul only, because almost all dead trees are removed annually and ground for use in power generation (Zach Jorgensen, City of St. Paul Forestry Division, personal communication), we assumed that 100% of carbon stored in trees would be emitted to the atmosphere within a year of death. On the other hand, trees on forest, agricultural or undeveloped land outside the city typically remain intact and experience natural decomposition as little forestry management exists there. For these sites, we estimated carbon releases from decomposition in two ways: (1) delayed release from tree roots; (2) delayed release from aboveground biomass. For the former, based on existing studies, we assumed and estimated belowground biomass to decompose over 20 years [35] with 20% of carbon released in the first year [60]. For aboveground biomass, we modified an exponential model of annual biomass loss following Olson et al. (1963) [61]:

$$C_{t}=0.5*M\;*\;e^{-kt}$$ (2)

Where

*C*_*t*_ = weight of carbon left at time t (kgC)
*M* = initial aboveground biomass before decomposition (kgC)
*k* = decomposition rate constant
0.5 = carbon concentration

Determining the value of *k* is a key step in this process. Decomposition rates vary by vegetation component and species which influences the timing and magnitude of carbon released [62]. It is generally assumed that the decay rate of logs is much lower than the decay rate of foliage and branches [63]. However, small twigs and branches do not always decay more rapidly than larger materials, depending mainly on site moisture content [63]. In addition to temperature and moisture, other factors that influence decay rates include soil nutrient content and microbial community [64,65]. Since regional climate plays a major role in determining the decay rate of dead wood [66], we searched for past studies that assessed the value of *k* in Minnesota. Although decay rate constants for many tree species were available in different geographic locations, little research existed that estimated species-specific decay rates for Minnesota specifically [66–68]. Therefore, to simplify calculations, an average decomposition rate constant was used for all species, without differentiating decay rates between logs, foliage and twigs. Specifically, we followed a large wood decomposition study from a temperate mixed forest ecosystem in north-central Minnesota and set *k* at 0.062 [67]. To compute the weight of carbon remaining, we multiplied the final biomass at time *t* by the percent carbon content, which is reported to remain at 50% throughout the decomposition process, and to be relatively constant among sites and species [69]. Lastly, for trees outside the city of St. Paul located on other land uses (e.g., developed land), which are typically chipped and applied as landscape mulch, we conservatively assumed that 80% of carbon was released in the first year following mortality [70].

Tree growth rate should also be considered in estimating carbon sequestration rates as this is critical to carbon sequestration. Using values from a northeastern US study which estimated growth rates based on individual tree species group [71], we identified the following average dbh growth rates: aspen/alder/cottonwood/willow, 0.41 cm/year; spruce and pine, 0.35 cm/year; maple/oak/hickory/beech, 0.33 cm/year; other hardwood, 0.29 cm/year; cedar/larch, 0.19 cm/year; and soft maple/birch, 0.15 cm/year. For trees surviving into the next year, the average observed annual growth by species was added to the tree dbh from the LiDAR-generated tree data to estimate tree dbh in year two. We then recalculated the biomass equation using the new dbh to identify carbon storage in trees in year two. We calculated annual carbon sequestration due to tree growth as the difference between carbon storage estimates for year 1 and 2.

Tree planting also warrants consideration in our estimates as planted trees sequester carbon. The City of St. Paul’s Forestry Unit oversees the majority of tree planting within city limits. We assumed that 15% of dead trees were replaced with trees of the same size and species in St. Paul based on the Forestry Unit’s current management practices (Zach Jorgensen, City of St. Paul Forestry Division, personal communication). We used a conservative 10% replanting rate for developed land in other jurisdictions, assuming that some of them may not replant to the extent that St. Paul does, and a 0% replanting rate for undeveloped land, given that little forestry management occurs on such land.

Lastly, estimates of the supply of carbon storage and sequestration were aggregated from the individual plant level to the census tract level in order to facilitate comparisons with subsequent demand assessments. The final equations for calculating annual net carbon sequestration are as follows:

$$C_{s}=C_{g}-C_{l}+C_{p}$$ (3)

$$C_{g}=0.5*\sum \left(B_{i,x+1}-B_{i,x}\right)=0.5*\sum e^{\beta_{0}+\beta_{1}*\ln(dbh_{i}+\Delta dbh_{i})}-e^{\beta_{0}+\beta_{1}*\ln(dbh_{i})}$$ (4)

$$C_{l}=\left\{\begin{array}{c} 0.5*\sum B_{u}*100\%\\ 0.5*\left\{\sum [B_{above,v}*\left(1-e^{-k}\right)]+\sum B_{below,v}*20\%\right\}\\ 0.5*\sum B_{w}*80\% \end{array}\right\}$$ (5)

$$C_{p}=0.5*\sum B_{j}$$ (6)

Where

*C*_*s*_ = net annual carbon sequestration per census tract (kgC)
*C*_*g*_ = total carbon gained from tree growth in year *x+1* (kgC)
*C*_*l*_ = total carbon lost from tree decomposition in year *x+1* (kgC)
*C*_*p*_ = total carbon gain from tree replanting in year *x+1* (kgC)
*i* = identifier for a single living tree for year *x+1*
*B*_*i*, *x*_ = total biomass for tree *i* in year *x* (kgC)
*B*_*i*, *x+1*_ = total biomass for tree *i* in year *x+1* (kgC)
*u* = identifier for a single tree that died in a year in St. Paul
*v* = identifier for a single tree that died in a year outside St. Paul on forest, agricultural or undeveloped land
*w* = identifier for a single tree that died in a year on undeveloped land uses
*B*_*u*_ = total biomass for tree *u* (kgC)
*B*_*above*, *v*_ = aboveground biomass for tree *v* (kgC)
*B*_*below*, *v*_ = belowground biomass for tree *v* (kgC)
*B*_*w*_ = total biomass for tree *w* (kgC)
*j* = identifier for a single tree planted in year *x+1*
*B*_*j*_ = total biomass for tree *j* (kgC)

### E. Estimating demand for carbon sequestration

Demand for carbon storage and sequestration service is expressed based on local anthropogenic CO_2_ emissions. Our fundamental assumption in measuring demand is that increasing CO_2_ emissions are associated with a greater need for carbon storage and sequestration service to mitigate climate change and that this need is an indicator of demand for this service. We quantify this need by estimating total carbon emissions by census tract, a level at which sufficient data exist and that can support policy formation. We compare demand estimated in this way to carbon sequestration supply estimated above to identify the ability of local trees to offset emissions. This thus indicates the degree of balance in supply and demand for this service.

We estimated CO_2_ emissions by sector. The Minnesota Pollution Control Agency (MPCA) groups emissions of carbon dioxide in Minnesota into seven sectors: agricultural, commercial, electric utility, industrial, residential, transportation and waste [72]. Average emissions intensity and energy consumption values for Minnesota were used in this estimation due to a lack of local-level data [72]. Estimated values were combined with information on population, number of vehicles, number of employees in industrial and commercial occupations and areas of agricultural land to calculate final demand by census tract (Table 2).

**Table 2:** Symbols and parameters used in carbon sequestration demand calculation (after MPCA, 2012 [72]).

Source	Time	Scale	Symbol	Parameter
MPCA	2008	state	*I*_*e*_	lbsCO_2_/kWh consumed
	2008	state	*I*_*t*_	Short Tons CO_2_/vehicle
	2008	state	*I*_*a*_	lbsCO_2_/acres harvested
	2008	state	*I*_*i*_	lbsCO_2_/ industrial employee
	2008	state	*I*_*r*_	lbsCO_2_/capita
	2008	state	*I*_*c*_	lbsCO_2_/commercial employee
	2008	state	*I*_*w*_	lbsCO_2_ emitted from wastewater treatment/capita
	2008	state	*E*	kWh consumed per year/capita
Generalized Land Use 2010 for the TCMA	2010	1:3000–1:1500	*A*_*a*_	Agricultural land in m^2^
U.S. Census 2007–2011 American Community Survey 5-Year Estimates	2007–2011	census tract	*P*	Number of people/census tract
	2007–2011	census tract	*W*_*i*_	Number of employees in industrial sectors
	2007–2011	census tract	*W*_*c*_	Number of employees in commercial sectors
2008–2012 American Community Survey 5-Year Estimates	2008–2012	census tract	*V*	Number of vehicles/census tract

Our estimates of carbon emissions from the electric utility sector exclusively account for the combustion of nonrenewable energy sources, given that renewable energy sources, such as geothermal, hydro, wind and solar, emit nearly zero carbon. Based on Xcel Energy, the utility company that serves most of the study area, 70% of the electricity in the study area comes from natural gas and coal combustion [73]. Accordingly, we calculated carbon sequestration demand from this sector as follows:

$$D_{e}=I_{e}\;*\;E\;*\;P\;*\;0.7\;*\;0.453592$$ (7)

Where

*D*_*e*_ = Carbon sequestration demand from electric utility sector (kgCO_2_)
*I*_*e*_ = Electric intensity measured as lbsCO_2_/kWh consumed based on MPCA estimates
*E* = Energy consumed as kWh/year/capita
*P* = Number of people/census tract
0.70 adjusts total emissions to account for the percentage of electricity from nonrenewable energy sources (e.g. natural gas, coal-fired plants)
0.453592 is a conversion factor that coverts lbs. to kg

Our estimate of carbon emissions from transportation is based on the number of vehicles (i.e., cars, trucks and vans) in a census tract used by workers 16 years of age and over from the 2008–2012 American Community Survey 5-Year estimates. We multiplied this by the amount of CO_2_ emitted per vehicle:

$$D_{t}=I_{t}\;*\;V\;*\;907.185$$ (8)

Where

*D*_*t*_ = Carbon sequestration demand from transportation sector (kgCO_2_)
*I*_*t*_ = Transportation intensity measured as short ton CO_2_/vehicle based on MPCA estimates
*V* = Number of vehicles /census tract
907.185 is a conversion factor that coverts short ton to kg

To estimate the demand for carbon sequestration from industrial and commercial sectors, we used total employment and carbon dioxide emission rates for each sector. Based on the 2011 U.S. American Community Survey, employment in industry included occupations in mining, construction, manufacturing, and transportation and warehousing. Likewise, wholesale trade, retail trade, information, finance, insurance, real estate rental and leasing, management, educational services, as well as entertainment, accommodation and food services jobs were grouped into commercial employment. We obtained the number of employees from the above occupations from 2011 US Census American Community Survey and used it to estimate carbon emissions from these sectors as follows:

$$D_{i}=I_{i}\;*\;W_{i}\;*\;0.453592$$ (9)

Where

*D*_*i*_ = Carbon sequestration demand from industrial sector (kgCO_2_)
*I*_*i*_ = Industrial intensity measured as lbsCO_2_/ industrial employee based on MPCA estimates
*W*_*i*_ = Number of employees in industrial sector
0.453592 is a conversion factor that coverts lbs. to kg

$$D_{c}=I_{c}\;*\;W_{c}\;*\;0.453592$$ (10)

Where

*D*_*c*_ = Carbon sequestration demand from commercial sector (kgCO_2_)
*I*_*c*_ = Commercial intensity measured as lbsCO_2_/commercial employee based on MPCA estimates
*W*_*c*_ = Number of commercial sector employees
0.453592 is a conversion factor that coverts lbs. to kg

We used a 2010 land-use map for the TCMA available from the Twin Cities Metropolitan Council to calculate areas of agricultural land in each tract in m^2^ [74]. We then computed agricultural carbon emissions as:

$$D_{a}=I_{a}\;*\;A_{a}\;*\;0.000247105\;*\;0.453592$$ (11)

Where

*D*_*a*_ = Carbon sequestration demand from agricultural sector (kgCO_2_)
*I*_*a*_ = Agricultural intensity measured as lbsCO_2_/acre harvested based on MPCA estimates
*A*_*a*_ = Area of agricultural land in m^2^
0.000247105 is a conversion factor used to convert m^2^ to acre
0.453592 is a conversion factor that coverts lbs. to kg

Similarly, we estimated carbon demand from residential and waste sectors using the population of each census tract from the 2011 U.S. Census American Community Survey as follows:

$$D_{r}=I_{r}\;*\;P\;*\;0.453592$$ (12)

Where

*D*_*r*_ = Residential sector carbon sequestration demand (kgCO_2_)
*I*_*r*_ = Residential intensity measured as lbsCO_2_/capita from MPCA estimates
*P* = Census tract population
0.453592 is a conversion factor that coverts lbs. to kg

$$D_{w}=I_{w}\;*\;P\;*\;0.453592$$ (13)

Where

*D*_*w*_ = Waste sector carbon sequestration demand (kgCO_2_)
*I*_*w*_ = Waste intensity measured as lbsCO_2_ emitted from wastewater treatment/capita from MPCA estimates
*P* = Census tract population
0.453592 is a conversion factor that coverts lbs. to kg

Lastly, CO_2_ emissions from all sectors were summed to estimate the net demand, *D*_*net*_ (kgC), for the carbon sequestration service in each tract. Based on the composition of CO_2_, we used a factor of 0.2729 to determine the mass of emitted carbon:

$$D_{net}=\left(D_{e}+D_{t}+D_{i}+D_{c}+D_{a}+D_{r}+D_{w}\right)\;*\;0.2729$$ (14)

### F. Supply–to-demand ratio analysis

We followed a two-step process to compare estimated carbon sequestration supply to demand. Firstly, we mapped supply and demand for each tract to visualize spatial correspondence between supply and demand. Secondly, we estimated a supply-to-demand ratio for each tract and the full study area to indicate the ability of supply to meet demand. Although the supply of carbon sequestration was assessed at the individual tree level, census tracts were selected as the minimum mapping unit for the supply-to-demand ratio analysis because demand for carbon sequestration was estimated at this level. We used the following formula to calculate the supply-to-demand ratio for each tract:

$$R=S/D_{net}\;*\;100\%$$ (15)

Where

*R* = ratio of supply to demand for carbon sequestration
*S* = supply of carbon sequestration service in kgC/census tract/year
*D*_*net*_ = demand for carbon sequestration in kgC/census tract/year

## Results

### A. Urban trees characteristics

**Fig 3:**
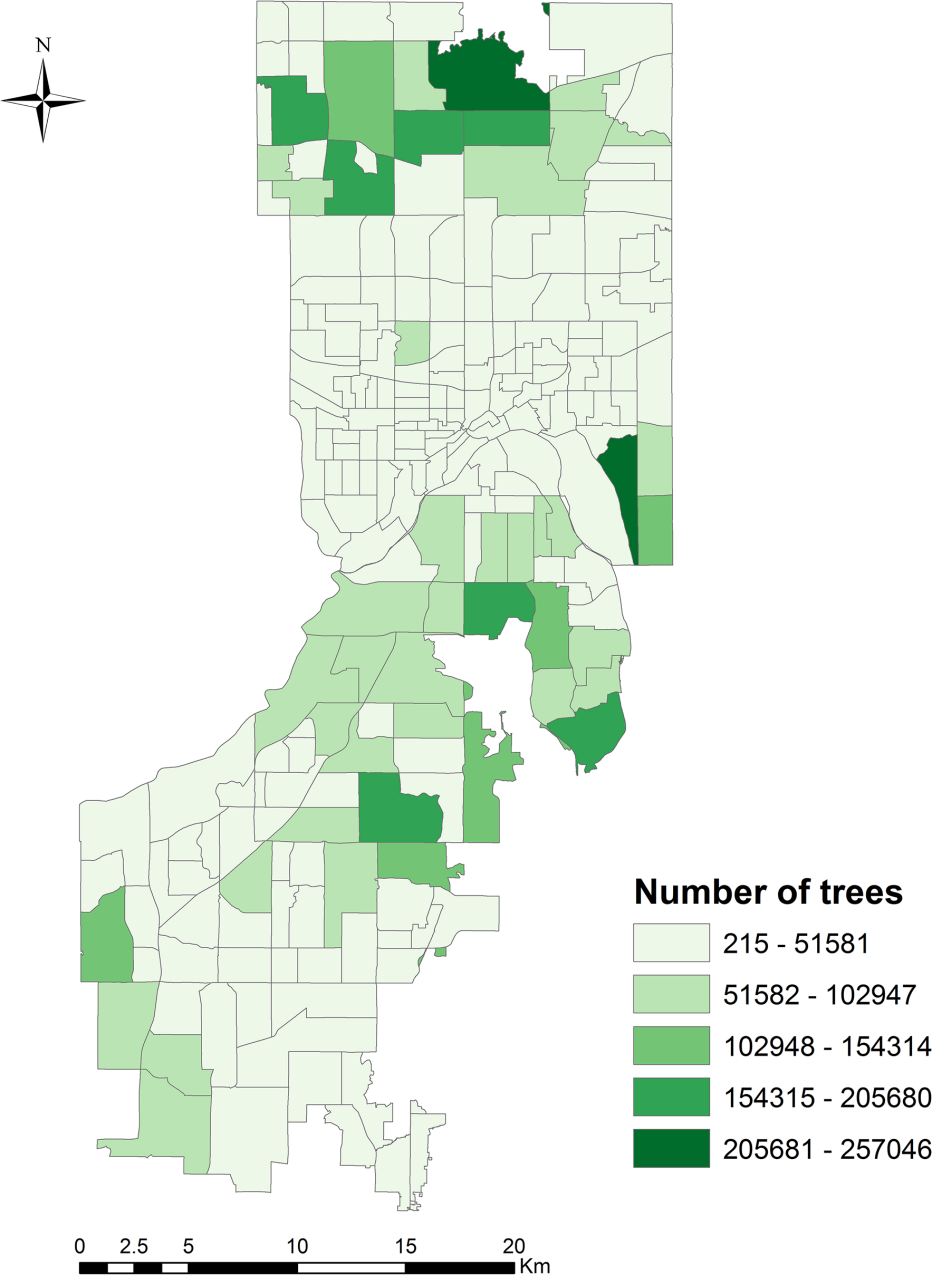
Number of urban trees by census tract for Dakota and Ramsey County, MN urban areas identified through LiDAR processing. A natural breaks (Jenks) classification system was used to more clearly represent trends in the data due to uneven distributions of values.

In all, LiDAR data processing identifies 7,291,140 trees in Dakota and Ramsey County urban areas. These trees are not evenly distributed by land-use type or region. Generally, northern Dakota County and northern Ramsey County have much denser tree canopy cover than the rest of the study area (Fig 3). The characteristics of trees also vary (Table 3). Overall, trees have an average height of 11.51 m (range: 3 m-440 m, SD = 4.62 m) and average crown width of 3.90 m (range: 2.6 m-16.93 m, SD = 1.07 m). Tree dbh ranges from 0.24 m-0.83 m (mean = 0.29 m, SD = 0.04 m). The LiDAR metric variable explains 76% of the variability in the crown width of trees (Adjusted *R* ^*2*^: 0.7559; *p* <0.001) (S1 Fig). The average bias between predicted and measured crown width is about 0.19 m, and the RMSE of the regression between them is 0.43 m (11% of the mean crown width).

**Table 3:** Statistics for individual trees identified via LiDAR processing in Dakota and Ramsey County, Minnesota.

	Tree height (m)	Crown width (m)	dbh (m)
Mean	11.51	3.90	0.29
Minimum	3.00	2.60	0.24
Maximum	440.00	16.93	0.83
Standard Deviation	4.62	1.07	0.04

### B. Carbon storage

**Fig 4:**
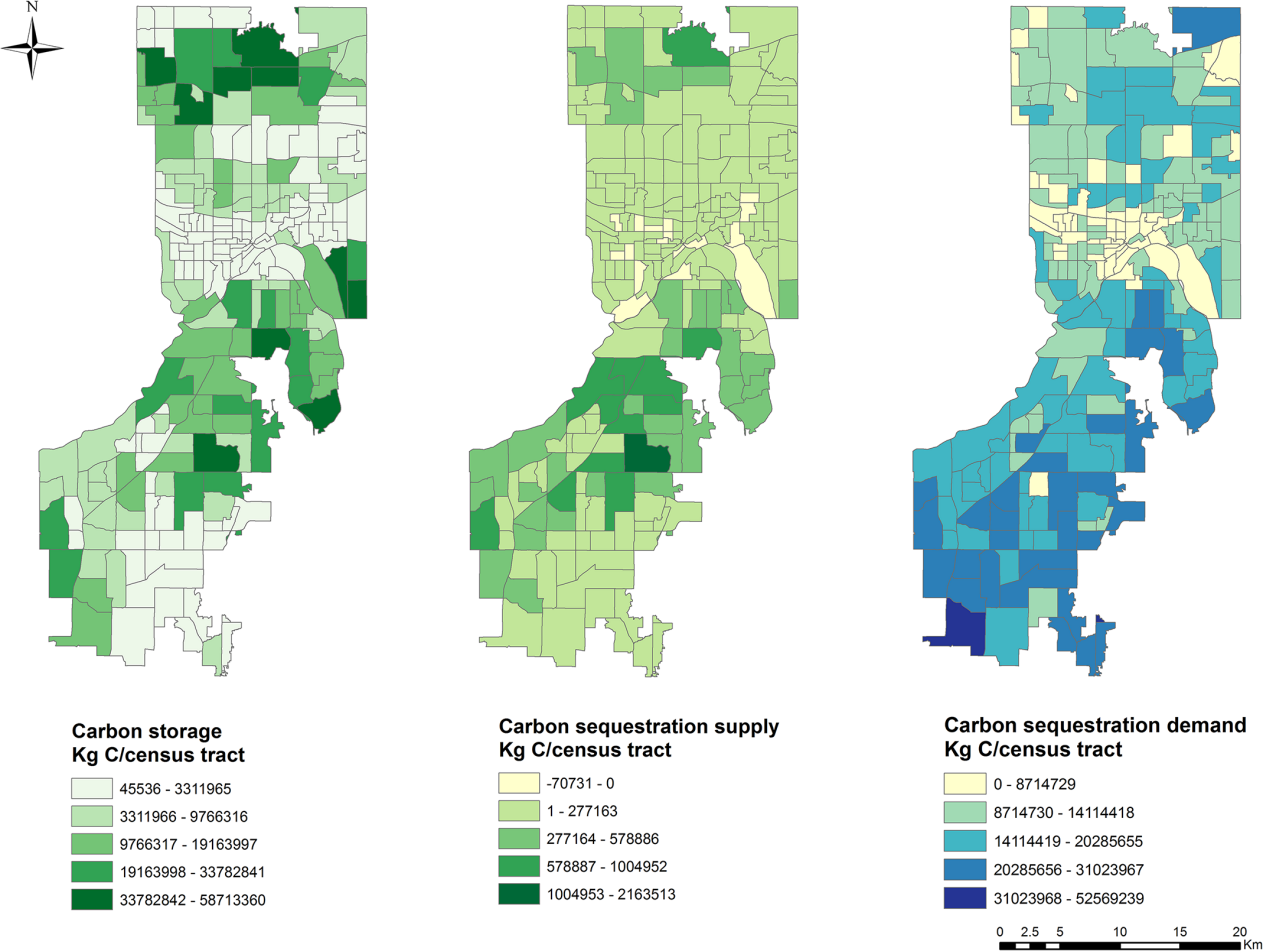
Carbon storage and carbon sequestration service supply and demand maps for Dakota and Ramsey County urban areas. A natural breaks (Jenks) classification system was used to more clearly represent trends in the data due to uneven distributions of values.

We estimate that urban tree resources in the study area store 1,735.69 million kgC of which 889.17 million kgC are stored by Dakota County trees and 846.52 million kgC are stored by Ramsey County trees. The carbon storage of individual trees ranges from 103.34 kg–3,402.61 kg. The average carbon storage per tree is 238.13 kg (range: 103.34 kg–3402.61 kg, SD = 106.81 kg, Table 4). Average carbon storage is 7.78 million kgC/census tract (range: 45,536 kg–58.71 million kg, SD = 10.64 million kg). This service is most abundant in northern Dakota County and in northern Ramsey County (Fig 4). In Dakota County, the highest carbon storage values occur in the county’s northeastern corner, where 43 million kgC/census tract are stored by urban trees. This service is also relatively high along the northern and western borders of Dakota County and gradually decreases to 14 million kgC/census tract when it passes through more central urban areas. The lowest service levels occur in agricultural southern townships.

**Table 4:** Statistics for carbon storage and sequestration service in urban areas in Dakota and Ramsey County, MN.

	Carbon sequestration (kgC /census tract/ year)		Carbon storage (kgC /census tract)	Carbon storage (kgC/tree)
	Supply (flow)	Demand	Supply	
Mean	149,897	13,845,718	7,783,356	238.13
Min.	-70,731	0	45,536	103.34
Max.	2,163,513	52,569,239	58,713,360	3,402.61
SD	254,434	6,711,763	10,637,338	106.81
Total	33,427,076	3087,595,137	1,735,688,447	1,735,688,447

In Ramsey County, this service decreases moving from north to south. The highest carbon storage levels occur in the northern region, where 58.7 million kgC/census tract are stored. Higher levels of carbon storage (mean = 22 million kgC) also occur in other areas of northern Ramsey County. This decreases to 7 million kgC/census tract moving towards central Ramsey County. The lowest levels occur in southern Ramsey County, where trees are more sparsely-distributed and store only 0.6 million kgC/census tract.

### C. Supply of carbon sequestration

Net annual carbon sequestration for the study area is estimated to be 33.43 million kgC/year. On average, 149,897 kgC/census tract are sequestrated by trees annually (Table 4). Annual carbon sequestered due to tree growth is 49.3 million kgC, and carbon storage gained from planting is 3.4 million kgC. Loss from tree mortality is 19.3 million kgC. The highest carbon sequestration service occurs in eastern Dakota County, where large forested parks exist (Fig 4) and trees capture 2.1 million kgC/year. Higher carbon sequestration levels occur in northern Ramsey County and in Dakota County where carbon captured by tree growth offsets carbon lost to tree mortality (mean = 0.7 million kgC/census tract/year, SD = 0.36 million kgC). Conversely, the carbon sequestration service is negative on some land in the St. Paul where trees are sparsely distributed and tree mortality and decay rates are higher.

### D. Demand for carbon sequestration

In total, 3087.60million kgC or 7.3% of total 2010 GHG emissions in the state of Minnesota (155.6 million CO2-equivalent tons [72]) are emitted annually through economic development and human consumption activities in the study area. The electric utility sector is the primary source of carbon emissions (1564.55 million kgC), followed by agriculture (618.69 million kgC), transportation (467.26 million kgC), residential (411.52 million kgC), waste (23 million kgC), industrial (2.23 million kgC) and commercial activities (0.33 million kgC). Moreover, commercial carbon emissions are concentrated in Ramsey County, while agricultural and industrial carbon emissions are distributed unevenly in Dakota County.

The spatial pattern exhibited by demand for carbon sequestration differs from that of supply (Fig 4). The highest demand occurs in southernmost Dakota County, where 55.2052.57 million kgC are emitted, but only 2596.83 kgC are sequestered. This emissions level is more than three times the average level for the overall study area (13.85 million kgC emissions/census tract). In general, greater demand occurs in southern Dakota County and northern Ramsey County, where census tracts have an average emission of 21.74 million kgC (SD = 4.20 million kgC). Most areas of lower demand occur in St. Paul (mean = 8.23 million kgC emissions/census tract, SD = 3.40 million kgC).

### E. Comparing supply with demand

A considerable mismatch exists between supply and demand for the carbon sequestration service in the study area (Fig 4). Firstly, a substantial carbon sequestration deficit exists as demand far exceeds the carbon sequestration supply in the area as a whole (3087.60million kgC/year versus 33.43 million kgC/year, Table 4). Secondly, with the exception of some census tracts in northern Dakota County where supply and demand are more coincident, a very high spatial mismatch occurs between demand and carbon sequestration by trees. A substantial undersupply exists in southern Dakota County in particular, where high demand is observed.

**Fig 5:**
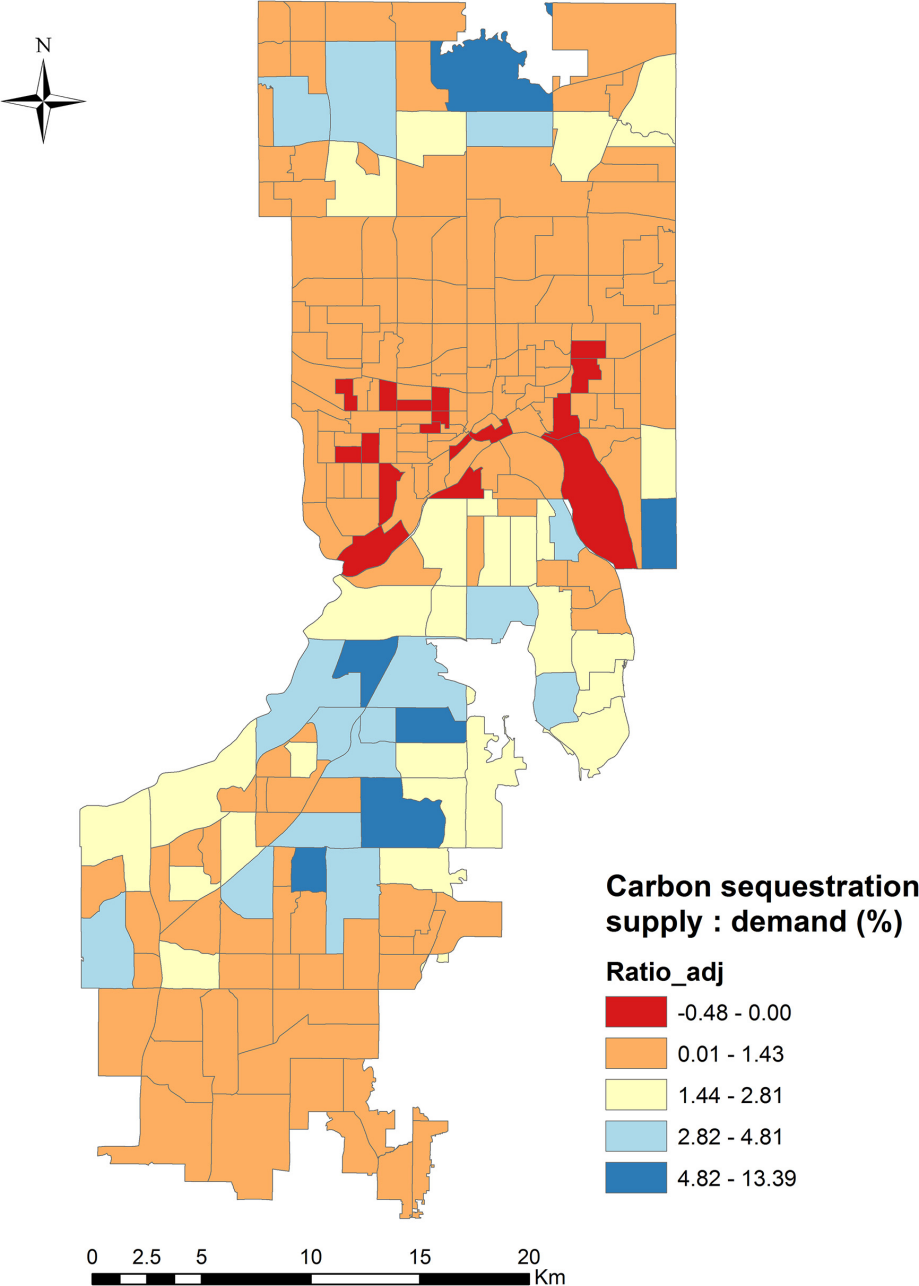
Carbon sequestration service supply-to-demand ratio map for Dakota and Ramsey County urban areas, representing the relative carbon sequestration balance. A natural breaks (Jenks) classification system was used to more clearly represent trends in the data due to uneven distributions of values. The first class was modified to show ratios below zero.

The magnitude of the difference between carbon sequestration supply and demand makes comparing them in absolute quantities challenging. The ratio map however, facilitates the comparison of supply with demand by demonstrating the relationship between them in a quantitative and easily-visualized manner (Fig 5). For example, the highest ratio occurs in north-central Dakota County where 13% of carbon emissions are sequestered by trees, approximately equaling the US average rate of GHG reduction provided by forest ecosystems (10%) [75]. Along the Mississippi River Corridor Critical Area (MMRRA) region of Dakota County, a heavily-forested area where 116 km of the Mississippi River and 21,853 hectares of adjacent corridor lands are designated and managed as natural areas, approximately 4% of demand is offset by trees. A similarly high ratio is observed in northern Ramsey County, where forested recreational areas cluster. Most of central Ramsey County and southern Dakota County, however, have a nearly zero carbon offset, with a ratio of 0.3%, less than half of the average ratio for the study area (0.9%, SD = 1.6%). Critically, supply-to-demand ratios below zero (-0.5%–0%) occur in some areas in St. Paul, indicating negative supply of carbon sequestration (i.e., that trees are releasing CO_2_).

## Discussion

Current assessments of ecosystem services tend to focus solely on the supply of these services. As this fails to consider a critical component of the ecosystem services dynamic, the demand or need for these services, these assessments do not fully represent the status of the services they assess. This strongly limits the use of these assessments in policy making as they fail to provide information on the full spectrum of ecosystem service delivery and consumption. Additionally, many existing studies are not conducted at scales relevant to policy making or lack a spatial component that could help policy makers identify the locations to which policies might be targeted or rely on data that are costly or difficult to acquire. In this study we presented a method for assessing both the supply and demand for an ecosystem service, carbon storage and sequestration by urban trees, as well as the relationship between this supply and demand, in a spatially explicit manner that could inform local policy making related to climate change mitigation. As such, this method begins to fill the existing gap in our ability to provide meaningful ecosystem service assessments to planners and policy makers. This method has numerous strengths that could link the ecosystem service more closely to planning and policy making as well as weaknesses that require further refinement.

### A. Strengths and limitations of the LiDAR-based method for assessing ecosystem service supply

The supply of carbon storage and sequestration depends on ecological functions and processes that take place at the individual plant level, including tree growth, mortality and decomposition. As such, estimating carbon storage and sequestration requires a method that goes beyond traditional proxy-based approaches that only consider the capacity of ecosystem service provision by land cover type (e.g., InVEST [76]). Our approach uses LiDAR-based techniques that capture biophysical attributes of individual trees and thus provides the information needed to quantify and map final carbon storage and sequestration supply at a finer scale by considering ecological functions and processes. As opposed to traditional forest inventory-based methods (e.g., i-Tree model [38]), our LiDAR-based method has additional advantages related to reduced need for field surveys and greater cost-effectiveness in terms of data capture and processing.

Our findings indicate that LiDAR is a reliable method for assessing urban tree features. We did not conduct direct validation of LiDAR-derived dbh and tree height due to a lack of field measurements. However, since previous studies indicate that the dbh-height-crown width relationship for a given set of species and site conditions is relatively stable [42], the high accuracy indicated by our assessment for estimated crown width suggests that our dbh estimates are also highly accurate. Our estimated RMSE for crown width (RMSE = 0.43 m) is also less than those of previous studies that estimated crown width based on biometric relationships with typical field-collected forest inventory variables (i.e., dbh, height, height-to-crown base, crown class, basal area per hectare, and trees per hectare) (RMSE: 0.6081–1.48 m) [77] and that derived crown width by delineating tree shapes using a local maximum algorithm on a canopy height model (RMSE = 1.36–1.41 m) [42]. This suggests that feature extraction approaches using LiDAR data such as the approach employed here are able to measure crown width with higher accuracy than these models.

The accuracy of LiDAR feature extraction depends not only on the algorithms used, but also the characteristics of the LiDAR dataset (e.g., number of returns, density) used as well as study site conditions (e.g., tree canopy density, number of vegetation layers, slope, and tree species composition). Previous studies have suggested that the error associated with LiDAR-derived forest variables can be reduced using high density LiDAR data and an accurate DTM [78]. Such data allow more laser pulses to penetrate the upper canopy and reach the ground. This, in turn, results in greater accuracy in information such as tree locations and attributes derived from such datasets. Thus the high accuracy of tree attribute measurements indicated by our low RMSE likely results at least in part from the use of a high-density LiDAR dataset in this study.

We find the accuracy of tree features to vary with location. For example, feature extraction accuracy is relatively lower for trees located along building edges or sparsely distributed on agricultural lands, where ground features with rounded shapes (e.g., water towers, ethanol refineries) may be misclassified as trees. The lowest accuracy of LiDAR processing is observed on water which tends to absorb laser pulses and thus returns very weak signals to LiDAR sensors. This indicates that visual interpretation and validation of LiDAR-generated urban forest data is critical to ensuring the accurate extraction of tree features. Specific attention should be given to trees identified as located in water, on agricultural lands and in dense urban areas with complex building structures.

The inability of our LiDAR-processing technique to identify individual tree taxa could impact the accuracy of our carbon storage and sequestration estimates. The generalized allometric models that we used to generate these estimates depend on information related to tree taxa. Because the LiDAR processing in this study does not generate species data for individual trees, we assigned taxa to individual trees via random sampling using estimates of the relative abundance of dominant tree species from field data. Inaccuracy in these taxon assignments would lead to error in the calculation of above-ground biomass which in turn could affect the accuracy of carbon storage and sequestration estimates. Mapping tree species using a fusion of multispectral/hyperspectral and LiDAR data, a promising technique for identifying individual tree species, could minimize such error [79]. Although this approach has been shown to be capable of identifying tree species, it is challenging and not yet fully developed in urban areas due to their great native and exotic species richness and great spatial variation in tree species occurrence [79]. Field sampling of tree species at a finer resolution would also improve LiDAR-based allometric modeling, but at considerable cost to labor and time. Future studies might also avoid error related to inaccurate tree species identification by using methods other than allometric models in estimating carbon storage and sequestration. For example, such studies might investigate the use of LiDAR metrics with locally-adapted linear and nonlinear regression to estimate carbon storage and sequestration. In so doing, these studies would seek to predict carbon storage and sequestration based upon different combinations of LiDAR-derived forest variables and might improve upon the accuracy of estimates.

As mentioned above, uncertainty and error exist in our forest structure estimates, for example, in the estimates of dbh and crown width as well as in the use of generalized allometric models, which may impact estimated carbon storage and sequestration. These biases, however, are only minimally quantified in this study due to a lack of direct monitoring data for carbon storage and sequestration supply. To better assess these biases, future studies could: (1) systematically collect and use field data on tree attributes to validate LiDAR results; (2) identify sources of error and quantify error in the final estimates of carbon storage and sequestration supply based upon error propagation theory [80] or (3) compare the supply map generated using this method with independent studies for the same study region.

### B. Implications for land-use planning and management

Quantifying the supply of carbon storage and sequestration by trees is increasingly important, given that such vegetation is a major terrestrial carbon pool that could help to mitigate climate change. Our assessment of carbon storage and sequestration by urban trees provides key insights that could inform climate change mitigation strategies at the local level, strategies that urban municipalities increasingly seek to implement. Climate change mitigation via GHG reduction, for example, is identified as a significant goal in the 2030 Comprehensive Plan for Dakota County and similar goals exist for cities worldwide, reflecting a growing interest in achieving carbon neutrality as a means to mitigate climate change.

Our assessment highlights the potential role of urban trees in reaching this carbon neutrality, indicating that urban forests can act as a carbon sink, albeit one that may be limited in terms of capacity. We find that trees in the study area store a significant amount of carbon (1735.69 million kgC). Our calculated carbon storage values per tree (mean = 238.13KgC/tree) are comparable to those estimated in a series of studies implemented in ten other US cities (mean = 227.00 kgC/tree, range: 91.81–638.95 kgC/tree) and are similar to estimates for densely-urbanized cities like New York, NY (235.07 kgC/tree), Philadelphia, PA (227.64 kgC/tree) and Boston, MA (244.97 kgC/tree) [35]. In line with other urban studies [44,81], our findings indicate that the direct net carbon sequestration by trees in urban areas makes only a small contribution to climate change mitigation (33.43 million kgC/year), offsetting approximately 1% of annual anthropogenic CO_2_ emissions in the study area (3758.12 million kgC/year). This carbon offset rate is slightly greater than the rate obtained in other studies (Shengyang, China: 0.26%; Barcelona, Spain: 0.47%) [44,45], a difference which may result from actual differences in urban forest structure among locations or from different methodologies. This low offset rate suggests that city governments should recognize that the supply of carbon storage and sequestration service by urban trees is limited and that they should seek to not only maintain such services, but also to reduce demand via emissions reductions. This is not to say that trees are unimportant in climate change mitigation especially as trees provide additional benefits that could assist in reducing emissions, for example, through shading and evapotranspiration, which can reduce energy consumption for heating and cooling leading to avoided CO_2_ emissions [82]. These additional benefits were not considered in this study.

Spatially explicit mapping is needed to quantify ecosystem services because the supply of and demand for ecosystem services vary geographically. The design and implementation of policies for achieving sustainability also depends on such spatially explicit information [83], which can improve the ability of planners and policy makers to target programs to locations where they may be most effective or needed [17]. The method demonstrated in this study makes the spatial variability of carbon storage and sequestration and its relationship with the underlying ecosystem components much clearer. The supply map our method produces illustrates how the supply of carbon storage and sequestration varies in space due to the spatial variation of urban tree resources (Fig 4). While large forested parks and reserves near MMRRA plays a major role in enhancing carbon storage, backyard and street trees within residential areas and trees on undeveloped land constitute a considerable proportion of the biomass carbon pool in more heavily urbanized areas. Spatial variability of carbon sequestration in urban trees mirrors that of storage, further indicating areas that are critical to the supply of this service. Such findings could help managers and policy makers to better understand where and how carbon storage and sequestration are provided in urban landscapes and to highlight the important role that urban tree protection ordinances and forest management practices can play in providing climate change mitigation benefits in urban areas.

Our method also facilitates the identification of spatially-explicit patterns in ecosystem service demand and the factors that influence these patterns. Our results indicate that demand for carbon sequestration is highest for the electricity utility sector, followed by the agricultural and transportation sectors. For example, in southern Dakota County and northern Ramsey County, with moderate populations, high vehicle numbers and more agricultural land, we find higher carbon emissions and thus greater demand (Fig 4). Conversely, lower demand for carbon sequestration occurs in highly urban St. Paul. This occurs because, although highly-urbanized census tracts have a high demand for carbon sequestration from residential, waste, industrial and commercial sectors, they have lower overall populations, vehicle numbers, and agriculture land areas than suburban areas with lower population densities and larger parcels. Additionally, although St. Paul is the most heavily urbanized part of the study area, important institutions and commercial and residential land uses also occur in other locations in the study area. This land use pattern further explains why most of St. Paul has relatively low carbon sequestration demand and why strategies for reducing demand might best be targeted to other locations. Our demand maps facilitate the identification of patterns such as these and can thus improve our understanding of the spatial variability in demand for ecosystem services and the factors that influence them. This in turn can improve our ability to identify and enact policies and management actions that enhance service delivery in the locations where demand is highest.

### C. The supply-to-demand ratio: an index of ecosystem service status

An important issue with ecosystem service assessments is that measures of ecosystem service supply fail to consider how this supply compares with demand and thus cannot indicate the true status of an ecosystem service [84]. Assessments that compare supply with demand, as the present study does, better indicate the level of balance between benefits supplied by ecosystems and the societal need for these benefits. The supply-to-demand ratio we demonstrate in this study quantifies this balance, identifying the level of carbon storage and sequestration delivered locally from ecosystems to socio-economic systems. A particular strength of this ratio lies in its spatial explicitness, which facilitates consideration of the spatial heterogeneity of ecosystem service supply and demand in identifying areas with supply- demand tensions [22,84,85]. A previous study has used the supply-to-demand ratio of water provisioning service to indicate the level of water scarcity, mapping water balances under different global change and mitigation scenarios [85]. Our supply-demand ratio could be used in a similar manner, for example, to inform and thus improve local-level policy making related to carbon storage and sequestration, a service that is delivered globally, via an analysis of the supply-demand ratio under different scenarios.

Our supply-to-demand ratio map provides a location-specific overview of the status of carbon storage and sequestration across an urban area (Fig 5). In so doing, it not only identifies locations where carbon sequestration supply and demand are in greater or lesser balance, but, with closer examination, also helps to reveal the underlying factors that influence this supply and demand (e.g., number of trees, population distribution, number of vehicles). For example, the highest supply-to-demand ratio occurs in the MMRRA region where trees are dominant landscape features (i.e., high supply) and both population densities and traffic volumes are low (i.e., low demand). Conversely, landscapes characterized by agricultural lands or sparsely-distributed trees (i.e., low supply) where heavier traffic or higher populations at lower densities concurrently exist (i.e., high demand) typically have smaller ratios. Since St. Paul has relatively fewer trees than the rest of the study area, and southern Dakota County has relatively more agricultural land and higher populations, both areas have smaller supply-to-demand ratios. Such information could help policy makers and land managers to identify location-specific mechanisms for improving supply-to-demand ratios.

Implementing climate change mitigation policy at the local level requires us to answer questions associated with where and how to reduce service deficits to achieve a supply-demand balance. As discussed above, analyses such as the one described here that explicitly examine the spatial balance in supply and demand provide insights that could help cities to identify and spatially target practices to achieve carbon neutrality. For example, maps such as those generated by this study highlight key locations where supply and demand are out of balance as well as locations where the ability of urban trees to provide carbon sequestration or demand are relatively high or low. This indicates locations to which tree planting or protection ordinances to enhance carbon storage and sequestration or policies that encourage the use of public transit might best be targeted to mitigate climate change. As planners and policy makers seek to reduce the demand for carbon storage and sequestration while increasing the supply of these services, the method for quantifying, mapping and comparing supply and demand for carbon storage and sequestration illustrated in this study opens up a broad avenue for integrating the notion of carbon neutrality with urban forest management and carbon emissions reductions in policy making. As such supply-demand ratio maps in combination with basic supply and demand maps have great potential to improve the quality of policy and management decisions.

Because ecosystems provide multiple services at a given point in time and space, it is important to recognize that tradeoffs may exist among different services with regard to their supply and demand. However, it is difficult to assess these tradeoffs as units for the supply and demand for different services vary, depending on the type and dimension of services considered and the way in which they are quantified. Our supply-to-demand ratio provides a straightforward and standardized way to facilitate trade-off assessments, because this ratio is standardized and unitless. Thus such ratios could be used to directly compare multiple ecosystem services. Such ratios have the added benefits of increasing flexibility in indicator selection, given that they do not require measures of supply and demand for different services to have the same unit or order of magnitude. When measures of service supply and demand are not directly comparable, they are typically compared using semi-quantified values assigned through processes like normalization, standardization or expert-based ranking [86]. For example, based on a literature review and expert opinions, Burkhard et al. (2012) translated the provision of ecosystem services into five categories classified by numerical values: 0 = no relevant capacity, 1 = very low, 2 = low, 3 = moderate, 4 = high, and 5 = very high [26]. In generating such ordinal values, one is forced to make judgements as to what level of service supply or demand falls into a given category. Such judgements have well-recognized impacts on resulting estimates. Using supply-demand ratios to assess and compare tradeoffs among different services alleviates this effect as it results in a standardized measure that does not require such value judgments. Our study assessed only one ecosystem service and did not analyze tradeoffs among multiple services using the supply-to-demand ratio in the manner suggested here. We suggest that future efforts examine these tradeoffs among ecosystem services using the supply-to-demand ratio. The identification of these spatial tradeoffs could serve to further unravel the mechanisms behind the interrelationships among multiple services.

### D. Caveats in assessing spatial mismatch between supply and demand

Special attention should be paid to the issue of scale when assessing in the balance between ecosystem service supply and demand spatially. The supply-to-demand ratio we demonstrate here could be implemented at any scale (spatial extent and grain), and the scale chosen should depend on the scale at which ecosystem services are produced, as well as the institutional scale at which they are received. The development of a spatial supply-demand ratio analysis for a given area of interest would first require identification of the SPA where ecosystem services are generated (e.g., population, ecosystem, landscape, biome, global) and the SBA where ecosystem services are demanded (e.g., individual, household, municipal, national, global) [87]. However, as ecosystem services are often supplied and consumed at multiple scales, identifying the proper scale for an analysis is challenging. For example, although carbon storage and sequestration is commonly taken as a global service, the supply of carbon storage and sequestration can be assessed at the plant, forest, landscape or global level. While beneficiaries of carbon storage and sequestration exist globally, demand for carbon sequestration occurs across local, regional and global scales and, indeed, can vary substantially with the scale and location considered. In identifying the best scale for an analysis one might consider the policy choices available and the scale at which they will operate, as well as the scale of information needed by policy makers and managers, who are often the end-users of ecosystem service assessments [88]. In our example, we sought to provide information illustrative of that required by policy makers within a metropolitan area. As such, we quantified carbon storage and sequestration service supply at the individual plant scale and aggregated this to the landscape scale for comparison with demand at a policy-relevant level. Future studies that use this methodology could aggregate to scales appropriate to specific planning and policy-making contexts. Additionally, although our study only considered a single scale in quantifying service demand, a multi-scale approach could be used to provide information of demand across multiple scales. For instance, demand for carbon sequestration could be assessed using participatory methods at the local scale [89], then analyzed using proxy or expert-based methods at the global scale [90]. Combining these methods would facilitate a wide range of ecosystem service assessments with purposes ranging from education to accounting for human well-being to specific landscape planning and management problems [91].

Linking supply and demand is another key issue in the assessment of ecosystem services. For ecosystem services with *in situ* spatial relationships (e.g., soil formation, erosion regulation) or proximity relationships (e.g., flood regulation, coastal protection) [4,92], spatial flow models that examine either the natural flow paths or anthropogenic flow corridors from SPA to SBA would be useful for exploring dynamic ecosystem service flows from supply sites to demand sites [10,13]. In this regard, processed-based models coupled with the ratio mapping method detailed here could be used to specifically identify service source, sink and benefit areas. Given carbon storage and sequestration is a singular service which is omnidirectional and spatially transferable, modeling the flow path of this service is rather challenging and not recommended. The spatial mismatch analysis presented in this study represents a simplified method for linking the supply of and demand for carbon storage and sequestration. This method focuses solely on the balance between carbon and sequestration service provision and demand within spatial units inside city boundaries and ignores the spatial import and export of carbon storage and sequestration at greater scales. Nevertheless, as discussed above, for a given region, and under the specific context of carbon neutrality and climate change mitigation, it is very useful and policy-relevant to assess the balance between the supply of and demand for this globally-delivered service in a spatially-explicit way at the local level.

## Conclusions

Locations of high ecosystem service supply are often poorly matched with locations of high service demand. Quantifying, mapping and comparing such areas is critical to enhancing the ability of ecosystem service assessments to inform policy making as such information can help policy makers to visualize and thus to better understand service provision in their jurisdictions. This study demonstrates an approach to assessing carbon storage and sequestration provided by urban forests that distinguishes among the supply and demand for this service in a spatially-explicit manner. Assessing carbon storage and sequestration in this way improves both our understanding of the ecological capacity of urban trees to store and sequester CO_2_ and our ability to consider relationships between this capacity and demand. In general, this spatially-explicit, supply-and-demand-driven approach to studying carbon storage and sequestration has great potential to improve assessments of this service in urban environments and the incorporation of such assessments into policy making associated with climate change mitigation and carbon neutrality in cities. This approach, if adapted for use with additional services, could additionally improve the general consideration of ecosystem services in local policy and land management decision making and the overall quality of environmental decision-making in local jurisdictions.

## Supporting Information

S1 Fig Simple linear regression between LiDAR-derived crown width and crown width measured from the orthophotograph. (TIF)

## References

[1] Millennium Ecosystem Assessment. Ecosystems and Human Well-being Washington, DC: Island Press; 2005 10.1196/annals.1439.003

[2] De Groot RS, Alkemade R, Braat L, Hein L, Willemen L. Challenges in integrating the concept of ecosystem services and values in landscape planning, management and decision making. Ecol Complex. Elsevier B.V.; 2010;7: 260–272. 10.1016/j.ecocom.2009.10.006

[3] Burkhard B, Kandziora M, Hou Y, Müller F. Ecosystem service potentials, flows and demands-concepts for spatial localisation, indication and quantification. Landsc Online. 2014;32: 1–32. 10.3097/LO.201434

[4] Fisher B, Turner RK, Morling P. Defining and classifying ecosystem services for decision making. Ecol Econ. Elsevier B.V.; 2009;68: 643–653. 10.1016/j.ecolecon.2008.09.014

[5] Haase D, Larondelle N, Andersson E, Artmann M, Borgström S, Breuste J, et al A quantitative review of urban ecosystem service assessments: concepts, models, and implementation. Ambio. 2014;43: 413–33. 10.1007/s13280-014-0504-0 24740614 PMC3989520

[6] De Groot RS, Wilson MA, Boumans RM. R. A typology for the classification, description and valuation of ecosystem functions, goods and services. Ecol Econ. 2002;41: 393–408. 10.1016/S0921-8009(02)00089-7

[7] Boyd J, Banzhaf S. What are ecosystem services? The need for standardized environmental accounting units. Ecol Econ. 2007;63: 616–626. 10.1016/j.ecolecon.2007.01.002

[8] Bastian O, Haase D, Grunewald K. Ecosystem properties, potentials and services–The EPPS conceptual framework and an urban application example. Ecol Indic. Elsevier Ltd; 2012;21: 7–16. 10.1016/j.ecolind.2011.03.014

[9] Bagstad KJ, Johnson GW, Voigt B, Villa F. Spatial dynamics of ecosystem service flows: a comprehensive approach to quantifying actual services. Ecosyst Serv. 2013;4: 117–125. 10.1016/j.ecoser.2012.07.012

[10] Serna-Chavez HM, Schulp CJE, van Bodegom PM, Bouten W, Verburg PH, Davidson MD. A quantitative framework for assessing spatial flows of ecosystem services. Ecol Indic. Elsevier Ltd; 2014;39: 24–33. 10.1016/j.ecolind.2013.11.024

[11] Wolff S, Schulp CJE, Verburg PH. Mapping ecosystem services demand: A review of current research and future perspectives. Ecol Indic. 2015;55: 159–171. 10.1016/j.ecolind.2015.03.016

[12] Schröter M, Barton DN, Remme RP, Hein L. Accounting for capacity and flow of ecosystem services: A conceptual model and a case study for Telemark, Norway. Ecol Indic. 2014;36: 539–551. 10.1016/j.ecolind.2013.09.018

[13] Villamagna AM, Angermeier PL, Bennett EM. Capacity, pressure, demand, and flow: A conceptual framework for analyzing ecosystem service provision and delivery. Ecol Complex. 2013;15: 114–121. 10.1016/j.ecocom.2013.07.004

[14] Geijzendorffer IR, Martín-López B, Roche PK. Improving the identification of mismatches in ecosystem services assessments. Ecol Indic. 2015;52: 320–331. 10.1016/j.ecolind.2014.12.016

[15] Willemen L, Verburg PH, Hein L, van Mensvoort MEF. Spatial characterization of landscape functions. Landsc Urban Plan. 2008;88: 34–43. 10.1016/j.landurbplan.2008.08.004

[16] Nelson E, Mendoza G, Regetz J, Polasky S, Tallis H, Cameron Dr, et al Modeling multiple ecosystem services, biodiversity conservation, commodity production, and tradeoffs at landscape scales. Front Ecol Environ. 2009;7: 4–11. 10.1890/080023

[17] Tallis H, Polasky S. Mapping and valuing ecosystem services as an approach for conservation and natural-resource management. Ann N Y Acad Sci. 2009;1162: 265–83. 10.1111/j.1749-6632.2009.04152.x 19432652

[18] Locatelli B, Imbach P, Vignola R, Metzger MJ, Hidalgo EJL. Ecosystem services and hydroelectricity in Central America: modelling service flows with fuzzy logic and expert knowledge. Reg Environ Chang. 2010;11: 393–404. 10.1007/s10113-010-0149-x

[19] Boumans R, Costanza R, Farley J, Wilson MA, Portela R, Rotmans J, et al Modeling the dynamics of the integrated earth system and the value of global ecosystem services using the GUMBO model. Ecol Econ. 2002;41: 529–560. 10.1016/S0921-8009(02)00098-8

[20] Villa F, Ceroni M, Bagstad K, Johnson G, Krivov S. ARIES (Artificial Intelligence for Ecosystem Services): A new tool for ecosystem services assessment, planning, and valuation. 11th annual BIOECON conference on economic instruments to enhance the conservation and sustainable use of biodiversity. Venice, Italy; 2009.

[21] Paracchini ML, Zulian G, Kopperoinen L, Maes J, Schägner JP, Termansen M, et al Mapping cultural ecosystem services: A framework to assess the potential for outdoor recreation across the EU. Ecol Indic. Elsevier Ltd; 2014;45: 371–385. 10.1016/j.ecolind.2014.04.018

[22] Stürck J, Poortinga A, Verburg PH. Mapping ecosystem services: The supply and demand of flood regulation services in Europe. Ecol Indic. 2014;38: 198–211. 10.1016/j.ecolind.2013.11.010

[23] Locatelli B, Imbach P, Wunder S. Synergies and trade-offs between ecosystem services in Costa Rica. Environ Conserv. 2013;41: 27–36. 10.1017/S0376892913000234

[24] Schulp CJE, Lautenbach S, Verburg PH. Quantifying and mapping ecosystem services: Demand and supply of pollination in the European Union. Ecol Indic. 2014;36: 131–141. 10.1016/j.ecolind.2013.07.014

[25] Nedkov S, Burkhard B. Flood regulating ecosystem services-mapping supply and demand, in the Etropole municipality, Bulgaria. Ecol Indic. 2012;21: 67–79. 10.1016/j.ecolind.2011.06.022

[26] Burkhard B, Kroll F, Nedkov S, Müller F. Mapping ecosystem service supply, demand and budgets. Ecol Indic. 2012;21: 17–29. 10.1016/j.ecolind.2011.06.019

[27] Van Berkel DB, Verburg PH. Spatial quantification and valuation of cultural ecosystem services in an agricultural landscape. Ecol Indic. 2014;37: 163–174. 10.1016/j.ecolind.2012.06.025

[28] Plieninger T, Dijks S, Oteros-Rozas E, Bieling C. Assessing, mapping, and quantifying cultural ecosystem services at community level. Land use policy. 2013;33: 118–129. 10.1016/j.landusepol.2012.12.013

[29] Casado-Arzuaga I, Madariaga I, Onaindia M. Perception, demand and user contribution to ecosystem services in the Bilbao Metropolitan Greenbelt. J Environ Manage. 2013;129: 33–43. 10.1016/j.jenvman.2013.05.059 23792888

[30] Syrbe R-U, Walz U. Spatial indicators for the assessment of ecosystem services: Providing, benefiting and connecting areas and landscape metrics. Ecol Indic. Elsevier Ltd; 2012;21: 80–88. 10.1016/j.ecolind.2012.02.013

[31] Liquete C, Zulian G, Delgado I, Stips A, Maes J. Assessment of coastal protection as an ecosystem service in Europe. Ecol Indic. Elsevier Ltd; 2013;30: 205–217. 10.1016/j.ecolind.2013.02.013

[32] Egoh B, Reyers B, Rouget M, Richardson DM, Le Maitre DC, van Jaarsveld AS. Mapping ecosystem services for planning and management. Agric Ecosyst Environ. 2008;127: 135–140. 10.1016/j.agee.2008.03.013

[33] Pataki DE, Alig RJ, Fung AS, Golubiewski NE, Kennedy CA, Mcpherson EG, et al Urban ecosystems and the North American carbon cycle. Glob Chang Biol. 2006;12: 2092–2102. 10.1111/j.1365-2486.2006.01242.x

[34] McPherson E, Nowak D, Rowntree R. Chicago’s urban forest ecosystem: results of the Chicago Urban Forest Climate Project USDA For Serv 1994;186.

[35] Nowak DJ, Crane DE. Carbon storage and sequestration by urban trees in the USA. Environ Pollut. 2002;116: 381–9. 11822716 10.1016/s0269-7491(01)00214-7

[36] Nowak DJ. Atmospheric carbon reduction by urban trees. J Environ Manage. 1993; 207–217.

[37] Nowak DJ, Greenfield EJ, Hoehn RE, Lapoint E. Carbon storage and sequestration by trees in urban and community areas of the United States. Environ Pollut. 2013;178: 229–36. 10.1016/j.envpol.2013.03.019 23583943

[38] Forest Inventory and Analysis National Program (FIA) [Internet]. 2014 [cited 10 Feb 2015]. Available: www.fia.fs.fed.us

[39] Nowak DJ, Crane DE, Stevens JC, Ibarra M. Brooklyn’s urban forest USDA For Serv 2002;290.

[40] Phillips DL. Assessment of ecosystem services provided by urban trees: public lands within the urban growth boundary of Corvallis, Oregon. City. 2011; 1–18.

[41] Myeong S, Nowak DJ, Duggin MJ. A temporal analysis of urban forest carbon storage using remote sensing. Remote Sens Environ. 2006;101: 277–282. 10.1016/j.rse.2005.12.001

[42] Ã SCP, Popescu SC. Estimating biomass of individual pine trees using airborne lidar. Biomass and Bioenergy. 2007;31: 646–655. 10.1016/j.biombioe.2007.06.022

[43] Brown S. Measuring carbon in forests: current status and future challenges. Environ Pollut. 2002;116: 363–72. 11822714 10.1016/s0269-7491(01)00212-3

[44] Liu C, Li X. Carbon storage and sequestration by urban forests in Shenyang, China Urban For Urban Green. Elsevier GmbH.; 2012;11: 121–128. 10.1016/j.ufug.2011.03.002

[45] Baró F, Chaparro L, Gómez-Baggethun E, Langemeyer J, Nowak DJ, Terradas J. Contribution of ecosystem services to air quality and climate change mitigation policies: the case of urban forests in Barcelona, Spain. Ambio. 2014;43: 466–79. 10.1007/s13280-014-0507-x 24740618 PMC3989519

[46] Jenkins J, Chojnacky D. National-scale biomass estimators for United States tree species. For Sci. 2003;49.

[47] Berland A. Twin Cities urbanization and implications for urban forest ecosystem services University of Minnesota 2012.

[48] US Census Bureau. 2010 Census Urban and Rural Classification and Urban Area Criteria [Internet]. 2010.

[49] Minnesota Geospatial Information Office [Internet]. 2015 [cited 1 Jan 2015]. Available: http://www.mngeo.state.mn.us/

[50] Overwatch [Internet]. 2015. Available: http://www.geospatial.overwatch.com/

[51] ESRI ArcGIS v.10.1 [Internet]. 2012. Available: http://www.esri.com/

[52] Opitz D, Blundell S, Rajendran R, Morris M. An approach for collection of geospecific 3D features from terrestrial LiDAR. ASPRS 2008 Annual Conference. Portland, Oregon; 2008. pp. 0–7.

[53] Minnesota Native Big Tree registry [Internet]. MNDNR; 2015. Available: http://www.dnr.state.mn.us/trees_shrubs/bigtree/index.html

[54] Jenkins J, Chojnacky D, Heath L, Birdsey R. Comprehensive database of diameter-based biomass regressions for North American tree species USDA For Serv 2004;319.

[55] Cairns MA, Brown S, Helmer EH, Baumgardner GA. Root biomass allocation in the world’s upland forests. Oecologia. 1997;111: 1–11. 10.1007/s004420050201 28307494

[56] Brown S, Lugo A. The storage and production of organic matter in tropical forests and their role in the global carbon cycle. Biotropica. 1982;14: 161–187.

[57] Dixon R, Brown S. Carbon pools and flux of global forest ecosystems. Science (5144). 1994;263: 185–190. 17839174 10.1126/science.263.5144.185

[58] Iverson L, Brown S, Prasad A. Use of GIS for estimating potential and actual forest biomass for continental South and Southeast Asia Effects of Land-Use Change on Atmospheric CO2 Concentrations. New York: Springer Verlag New York, Inc.; 1994.

[59] Ecoregions of the United States [Internet]. USDA Forest Service; 2015. Available: http://www.fs.fed.us/rm/ecoregions/products/map-ecoregions-united-states/

[60] Scheu S, Schauermann J. Decomposition of roots and twigs: effects of wood type (beech and ash), diameter, site of exposure and macrofauna exclusion. Plant Soil. 1994; 13–24.

[61] Olson JS. Energy storage and the balance of producers and decomposers in ecological systems. Ecology. 1963;44: 322–331.

[62] Chambers JQ, Higuchi N, Schimel JP, Ferreira LV., Melack JM. Decomposition and carbon cycling of dead trees in tropical forests of the central Amazon. Oecologia. 2000;122: 380–388. 10.1007/s004420050044 28308289

[63] Harmon ME, Franklin JF, Swanson FJ, Sollins P, Gregory SV., Lattin JD, et al Ecology of coarse woody debris in temperate ecosystems. Adv Ecol Res. 1986;15: 302.

[64] Berendse F, Berg B, Bosatta E. The effect of lignin and nitrogen on the decomposition of litter in nutrient-poor ecosystems: a theoretical approach. Can J Bot. 1987;65: 1116–1120. 10.1139/b87-155

[65] Boddy L, Owens E, Chapela I, Road N, Boddy L, Owens E, et al Small scale variation in decay rate within logs one year after felling: effect of fungal community structure and moisture content. FEMS Microbiol Lett. 1989;62.

[66] Russell MB, Fraver S, Aakala T, Gove JH, Woodall CW, D’Amato AW, et al Quantifying carbon stores and decomposition in dead wood: A review. For Ecol Manage. Elsevier B.V.; 2015;350: 107–128. 10.1016/j.foreco.2015.04.033

[67] David HA, Pastor J. Decomposition of aspen, spruce, and pine boles on two sites in Minnesota. Can J For Res. 1993;23: 1744–1749. 10.1139/x93-220

[68] Fraver S, Milo AM, Bradford JB, D’Amato AW, Kenefic L, Palik BJ, et al Woody debris volume depletion through decay: Implications for biomass and carbon accounting. Ecosystems. 2013;16: 1262–1272. 10.1007/s10021-013-9682-z

[69] Lambert RL, Lang GE, Reiners WA. Loss of mass and chemical change in decaying boles of a subalpine balsam fire forest. Ecology. 2014;61: 1460–1473.

[70] Mcpherson EG, Simpson JR. Through urban forestry: guidelines for professional and volunteer tree planters USDA For Serv 1999;171.

[71] Teck R, Hilt D. Individual-tree diameter growth model for northeastern United States USDA For Serv 1991;649.

[72] Minnesota Pollution Control Agency [Internet]. 2015. Available: http://www.pca.state.mn.us/

[73] Xcel Energy Inc. Power Generation supply [Internet]. 2013 [cited 7 Jan 2015]. Available: http://www.xcelenergy.com/Company/Operations/Power_Generation

[74] Minnesota Geographic Metadata. Metadata: Generalized Land Use 2010 for the Twin Cities Metropolitan Area [Internet]. 2011. Available: http://www.datafinder.org/metadata/GeneralizedLandUse2010.html

[75] The terrestrial carbon cycle: managing forest ecosystems U.S. EPA; 2007.

[76] Conte M, Nelson E, Carney K, Fissore C, Olwero N, Plantinga AJ, et al Terrestrial carbon sequestration and storage In: Kareiva P, Tallis H, Ricketts TH, Daily GC, Polasky S, editors. Natural capital theory and practice of mapping ecosystem services. New York, NY: Oxford University Press; 2011 pp. 111–128.

[77] Gill SJ, Biging GS, Murphy EC. Modeling conifer tree crown radius and estimating canopy cover. For Ecol Manage. 2000;126: 405–416. 10.1016/S0378-1127(99)00113-9

[78] Estornell J, Ruiz LA, Velázquez-Martí B, Fernández-Sarría A. Estimation of shrub biomass by airborne LiDAR data in small forest stands. For Ecol Manage. Elsevier B.V.; 2011;262: 1697–1703. 10.1016/j.foreco.2011.07.026

[79] Alonzo M, Bookhagen B, Roberts DA. Urban tree species mapping using hyperspectral and lidar data fusion. Remote Sens Environ. Elsevier Inc.; 2014;148: 70–83. 10.1016/j.rse.2014.03.018

[80] Romaguera M, Hoekstra AY, Su Z, Krol MS, Salama MS. Potential of using remote sensing techniques for global assessment of water footprint of crops. Remote Sens. 2010;2: 1177–1196. 10.3390/rs2041177

[81] Pataki DE, Carreiro MM, Cherrier J, Grulke NE, Jennings V, Pincetl S, et al Coupling biogeochemical cycles in urban environments: ecosystem services, green solutions, and misconceptions. Front Ecol Environ. 2011;9: 27–36. 10.1890/090220

[82] Simpson JR. Improved estimates of tree-shade effects on residential energy use. Energy Build. 2002;34: 1067–1076. 10.1016/S0378-7788(02)00028-2

[83] Maes J, Egoh B, Willemen L, Liquete C, Vihervaara P, Schägner JP, et al Mapping ecosystem services for policy support and decision making in the European Union. Ecosyst Serv. 2012;1: 31–39. 10.1016/j.ecoser.2012.06.004

[84] Paetzold A, Warren PH, Maltby LL. A framework for assessing ecological quality based on ecosystem services. Ecol Complex. Elsevier B.V.; 2010;7: 273–281. 10.1016/j.ecocom.2009.11.003

[85] Boithias L, Acuña V, Vergoñós L, Ziv G, Marcé R, Sabater S. Assessment of the water supply:demand ratios in a Mediterranean basin under different global change scenarios and mitigation alternatives. Sci Total Environ. Elsevier B.V.; 2014;470–471: 567–77. 10.1016/j.scitotenv.2013.10.003 24176705

[86] Schulp CJE, Burkhard B, Maes J, Van Vliet J, Verburg PH. Uncertainties in ecosystem service maps: a comparison on the European scale. PLoS One. 2014;9: e109643 10.1371/journal.pone.0109643 25337913 PMC4206275

[87] Hein L, van Koppen K, de Groot RS, van Ierland EC. Spatial scales, stakeholders and the valuation of ecosystem services. Ecol Econ. 2006;57: 209–228. 10.1016/j.ecolecon.2005.04.005

[88] Honey-Rosés J, Pendleton LH. A demand driven research agenda for ecosystem services. Ecosyst Serv. 2013;5: 160–162. 10.1016/j.ecoser.2013.04.007

[89] Palomo I, Martín-López B, Potschin M, Haines-Young R, Montes C. National Parks, buffer zones and surrounding lands: Mapping ecosystem service flows. Ecosyst Serv. 2013;4: 104–116. 10.1016/j.ecoser.2012.09.001

[90] Jacobs S, Burkhard B, Daele T Van, Staes J, Schneiders A. “The Matrix Reloaded”: A review of expert knowledge use for mapping ecosystem services. Ecol Modell. Elsevier B.V.; 2014;295: 21–30. 10.1016/j.ecolmodel.2014.08.024

[91] De Groot RS, Brander L, van der Ploeg S, Costanza R, Bernard F, Braat L, et al Global estimates of the value of ecosystems and their services in monetary units. Ecosyst Serv. Elsevier; 2012;1: 50–61. 10.1016/j.ecoser.2012.07.005

[92] Costanza R. Ecosystem services: multiple classification systems are needed. Biol Conserv. 2008;1: 8–10.

