# Supplementary figures and images for: Quantifying and Mapping the Supply of and Demand for Carbon Storage and Sequestration Service from Urban Trees
Source: PLoS One. 2015 Aug 28;10(8):e0136392. doi: 10.1371/journal.pone.0136392 (PMC4552758; doi:10.1371/journal.pone.0136392)

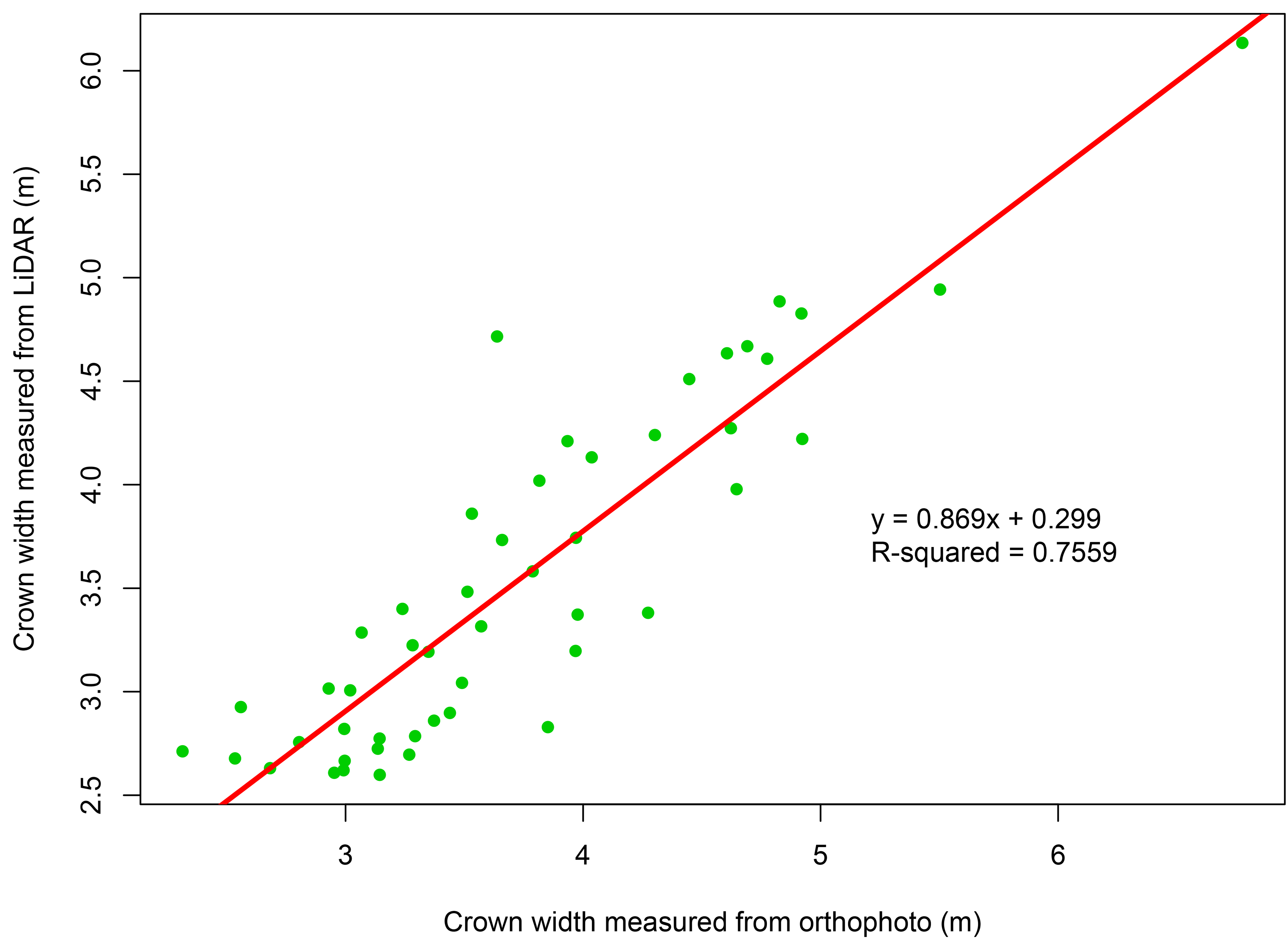

Supplement: S1 Fig — (TIF) [file pone.0136392.s001.tif]
